# Enzymatic synthesis of l-fucose from l-fuculose using a fucose isomerase from *Raoultella* sp. and the biochemical and structural analyses of the enzyme

**DOI:** 10.1186/s13068-019-1619-0

**Published:** 2019-12-05

**Authors:** In Jung Kim, Do Hyoung Kim, Ki Hyun Nam, Kyoung Heon Kim

**Affiliations:** 1 Department of Biotechnology, Korea University Graduate School, Seoul, 02841 South Korea; 2 Institute of Life Science and Natural Resources, Korea University, Seoul, 02841 South Korea

**Keywords:** l-Fucose, l-Fuculose, l-Fucose isomerase, *Raoultella*

## Abstract

**Background:**

l-Fucose is a rare sugar with potential uses in the pharmaceutical, cosmetic, and food industries. The enzymatic approach using l-fucose isomerase, which interconverts l-fucose and l-fuculose, can be an efficient way of producing l-fucose for industrial applications. Here, we performed biochemical and structural analyses of l-fucose isomerase identified from a novel species of *Raoultella* (*Rd*FucI).

**Results:**

*Rd*FucI exhibited higher enzymatic activity for l-fuculose than for l-fucose, and the rate for the reverse reaction of converting l-fuculose to l-fucose was higher than that for the forward reaction of converting l-fucose to l-fuculose. In the equilibrium mixture, a much higher proportion of l-fucose (~ ninefold) was achieved at 30 °C and pH 7, indicating that the enzyme-catalyzed reaction favors the formation of l-fucose from l-fuculose. When biochemical analysis was conducted using l-fuculose as the substrate, the optimal conditions for *Rd*FucI activity were determined to be 40 °C and pH 10. However, the equilibrium composition was not affected by reaction temperature in the range of 30 to 50 °C. Furthermore, *Rd*FucI was found to be a metalloenzyme requiring Mn^2+^ as a cofactor. The comparative crystal structural analysis of *Rd*FucI revealed the distinct conformation of α7–α8 loop of *Rd*FucI. The loop is present at the entry of the substrate binding pocket and may affect the catalytic activity.

**Conclusions:**

*Rd*FucI-catalyzed isomerization favored the reaction from l-fuculose to l-fucose. The biochemical and structural data of *Rd*FucI will be helpful for the better understanding of the molecular mechanism of l-FucIs and the industrial production of l-fucose.

## Background

l-Fucose (6-deoxy-l-galactose) is a rare sugar that occurs in a variety of living organisms from bacteria to humans [1]. For example, l-fucose is found in humans in the form of human milk oligosaccharides or glycoproteins, and microbial exopolysaccharides (EPSs) and seaweeds are often composed of l-fucose [2–6]. Due to various bioactive properties, l-fucose has the potential to be used as antiinflammatory, antitumor, and immune-enhancing drugs, as skin-whitening, skin-moisturizing, and anti-aging cosmetic agents, or as nutritional supplements [7–9].

For industrial production, l-fucose can be obtained via various routes that include extractive, chemical, and enzymatic methods. First, extraction of l-fucose from fucose-containing sources, such as seaweeds, plants, or animal tissues, has been attempted [1–6]. However, the process is laborious and costly due to the low content of l-fucose; for seaweed, the yields are often affected by the seasonal variation [2, 6]. Chemical synthesis of l-fucose can be achieved using a common sugar, such as d-galactose or d-mannose, as a raw material. However, this route is still considered impractical since multiple laborious steps are required, which produces low yields and requires high costs [10, 11]. Compared to these methods, enzymatic synthesis of l-fucose is more specific and environmentally friendly, and l-fucose may be produced more efficiently at a lower cost.

**Fig. 1:**
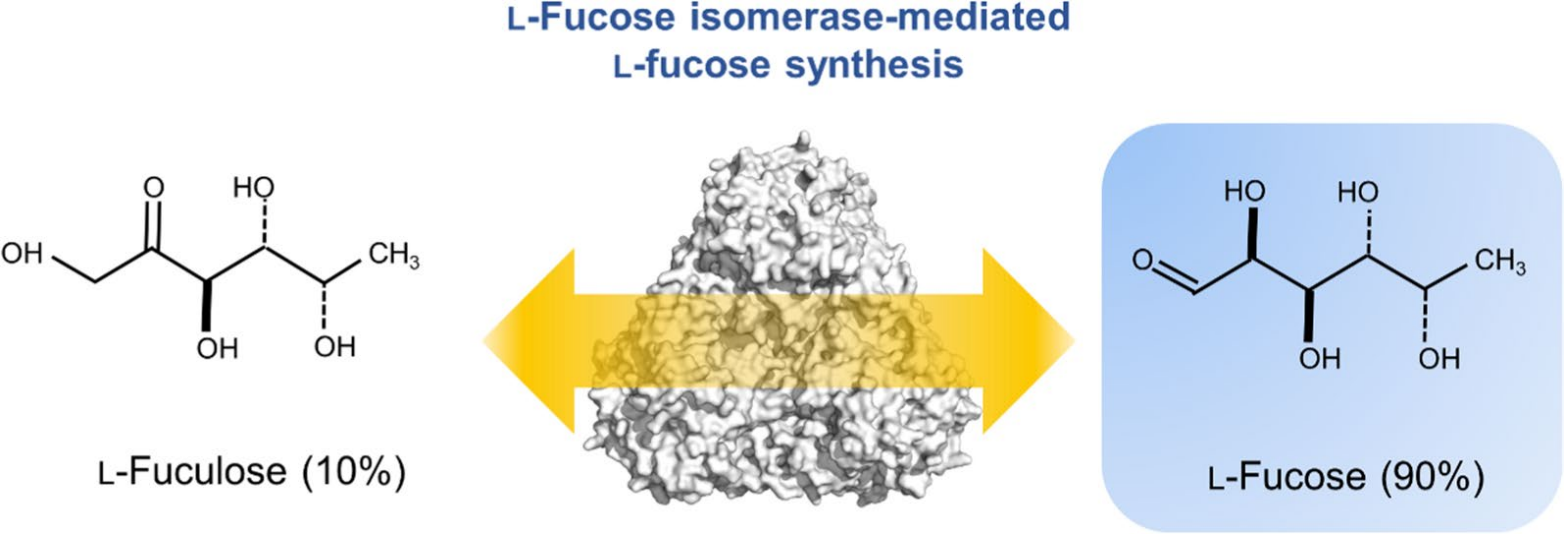
A schematic diagram for l-fucose synthesis from l-fuculose mediated by l-fucose isomerase

To date, two methods of enzymatically producing l-fucose from l-fuculose, the ketose form of l-fucose, have been developed. Although l-fuculose is more expensive and scarce in nature than l-fucose, l-fuculose can be synthesized as an intermediate from readily available resources, such as common sugars [12–14]. One approach is based on the reverse reaction for the l-fucose metabolic pathway, in which l-fuculose can be synthesized by an aldolase-catalyzed reaction between lactaldehyde and dihydroxyacetone phosphate (DHAP), followed by acid phosphatase-catalyzed dephosphatation [12]. Such an in vitro strategy can be further expanded to microbial fermentation using glucose as carbon source for l-fucose synthesis by constructing a metabolically engineered pathway. The other approach is chemicoenzymatic synthesis that employs d-galactose as the starting material from which l-fucitol is synthesized chemically in two steps, with l-fuculose enzymatically converted from l-fucitol by dehydrogenase [13, 15]. Both methods require l-fucose isomerase (l-FucI) (EC 5.3.1.25) to convert the intermediate l-fuculose to l-fucose. l-FucI is a type of ketol isomerase that catalyzes the interconversion of l-fucose and l-fuculose (Fig. 1) [16–19].

While l-fucose production using enzymatic methods is an attractive option, only a few l-FucIs (*Ec*FucI from *Escherichia coli* and *Kp*FucI from *Klebsiella pneumoniae*) have been examined concerning the equilibrium between l-fucose and l-fuculose. In *Ec*FucI and *Kp*FucI-catalyzed fucose isomerization, the reverse reaction is favored, and l-fucose is predominantly produced from l-fuculose [16, 19]. To increase the industrial applicability of l-FucIs for l-fucose synthesis, more detailed investigations into the equilibrium composition under various conditions and biochemical characterization using l-fuculose as the substrate are needed.

The genus *Raoultella* comprises gram-negative, aerobic, and non-motile bacteria belonging to the family *Enterobacteriaceae*, and includes four species, *R. electria*, *R. ornithinolytica*, *R. planticola*, and *R. terrigena* [20, 21]. The general habitats of these *Raoultella* species include natural environments, such as soil, water, and plants, but some strains may be present in the intestinal tract [22]. According to the National Center for Biotechnology Information (NCBI) database, genes associated with l-fucose utilization are commonly distributed among the four aforementioned *Raoultella* species.

We previously isolated a novel species in the genus *Raoultella* from abalone intestine (designated *Raoultella* sp. KDH14) and sequenced its full genome. Analysis and comparison of gene sequences identified an l-FucI from *Raoultella* sp. KDH14, which was designated as *Rd*FucI. In this study, we examined the conversion and equilibrium between l-fucose and l-fuculose using *Rd*FucI and performed the biochemical characterization and the structural analysis for *Rd*FucI. These will provide information of the fundamental understanding and possible industrial application of *Rd*FucI for the enzymatic synthesis of l-fucose.

## Results

### Bacterial isolation and *Rd*FucI identification

A colony that outgrew in the medium containing l-fucoidan sourced from *Laminaria Japonica* (Carbosynth, Compton, Berkshire, UK) as the sole carbon source was isolated from an abalone intestine harvested in South Korea (Additional file 1: Fig. S1). Comparison of the sequence identity based on 16S ribosomal RNA against the NCBI database revealed that the isolate was phylogenetically close to the members of the genus *Raoultella* (Additional file 1: Fig. S1). Thus, the isolated strain was identified as *Raoultella* sp. KDH14. After performing full genome sequencing, *Rd*FucI was identified from *Raoultella* sp. KDH14 on the basis of gene sequence identity.

*Rd*FucI is composed of 595 amino acids with a molecular mass of 65.5 kDa and an isoelectric point of 5.5. The basic local alignment search tool (BLAST) results indicated the high sequence identity of *Rd*FucI (> 90%) with other l-FucIs from various bacteria belonging to the families *Raoultella, Klebsiella,* and *Citrobacter*.

The identified *Rd*FucI was overproduced in *E. coli* BL21(DE3) with the N-terminal hexa-histidine tag and purified by his-tag affinity chromatography. When analyzed by sodium dodecyl sulfate-polyacrylamide gel electrophoresis (SDS-PAGE), the single band appeared around at 65 kDa, consistent with the calculated molecular mass of the monomer subunit.

### *Rd*FucI-catalyzed reaction favors l-fucose formation

**Fig. 2:**
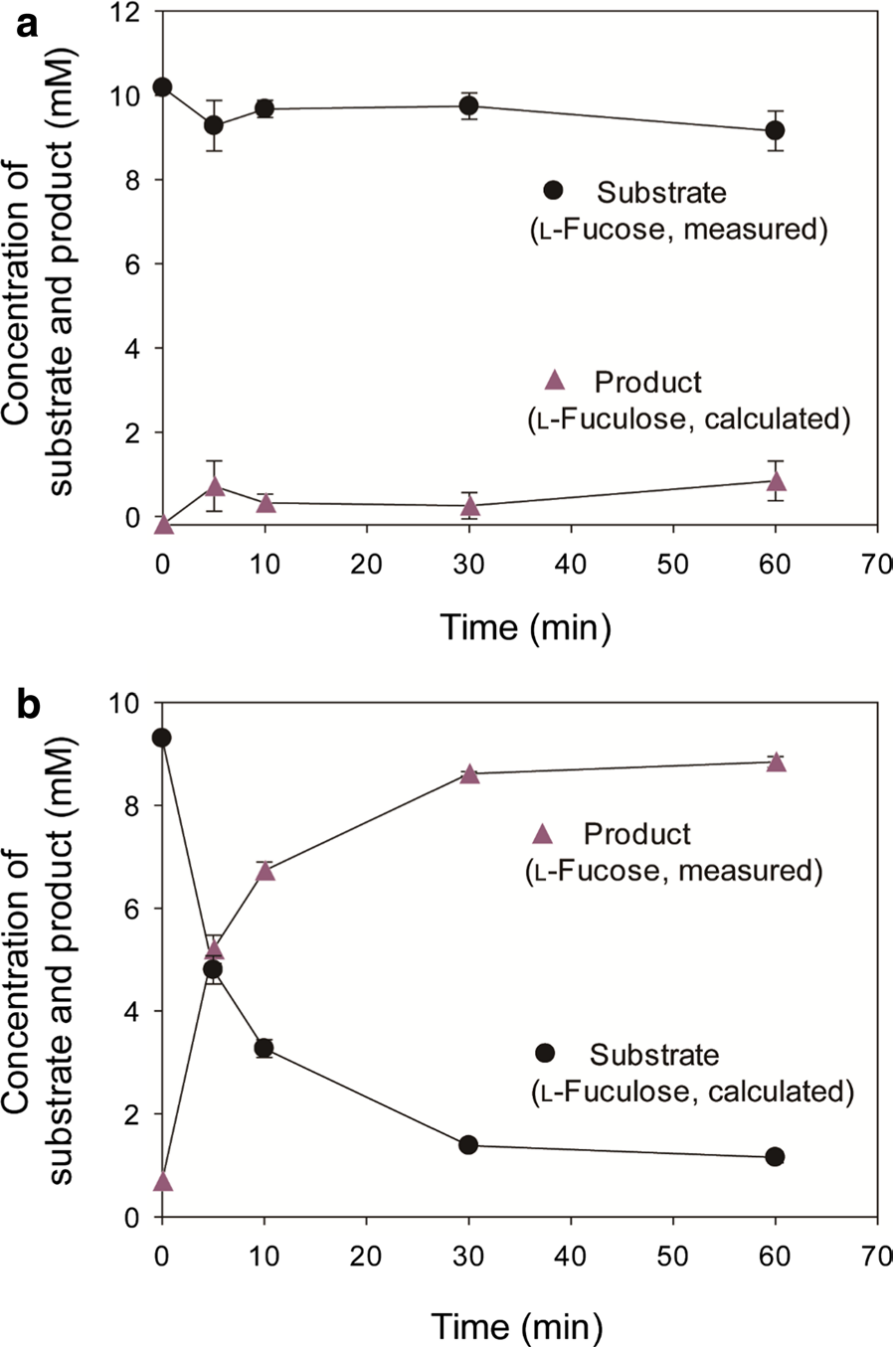
Enzymatic conversion of **a** l-fucose to l-fuculose (forward reaction) and **b** l-fuculose to l-fucose (reverse reaction) by *Rd*FucI. Enzymatic reaction was performed by 3 µg of *Rd*FucI with either **a** l-fucose or **b** l-fuculose at 10 mM as the starting substrate at 30 °C for 10 min in 20 mM sodium phosphate (pH 7.0) in the presence of 1 mM of Mn^2+^. l-Fucose concentration was experimentally measured and l-fuculose concentration was calculated by subtracting experimentally determined l-fucose concentration from total sugar concentration (10 mM). Experimental data represent means ± standard deviations of three replicates

To examine the isomerase activity of *Rd*FucI, the enzyme reaction was performed with l-fucose or l-fuculose as the substrate (Fig. 2). The enzyme activities for forward (l-fucose to l-fuculose) and reverse (l-fuculose to l-fucose) reactions were determined. Production of l-fuculose from l-fucose was confirmed by thin layer chromatography (TLC) and gas chromatography/mass spectrometry analysis (GC/MS) (Additional file 2: Fig. S2). In the interconversion between l-fucose and l-fuculose, there was no side product observed, meaning that one substrate yields one product (Additional file 2: Fig. S2). Thus, we consider it reasonable to use the calculated amount of l-fuculose concentration by experimentally measuring the amount of l-fucose.

The reverse reaction was 6.6 times faster than the forward reaction, and the specific activity for l-fuculose (63.9 U/mg) was higher than that for l-fucose (9.6 U/mg). In both reactions, the equilibrium ratio between l-fucose and l-fuculose, which was experimentally determined, was approximately 9:1, thus yielding the equilibrium constant (*K*_eq_) of  0.11. The value of *K*_eq_ was also theoretically determined as 0.23, based on the thermodynamic relation of the standard Gibbs free energy change of reaction and *K*_eq_ at equilibrium, $\mathop \Delta \limits_{r} G^{ \circ } = - RT\ln K_{\text{eq}}$, where *R* and *T* represent gas constant (8.314 J/mol K) and temperature (K), respectively. $\mathop \Delta \limits_{r} G^{ \circ }$ represents the standard Gibbs free energy change for the reaction of l-fucose to l-fuculose (0.859993 kcal/mol), which is listed in the database BioCyc (https://biocyc.org). There was some discrepancy between the experimental and theoretical values of *K*_eq_. A *K*_eq_ < 1 indicates that the reverse reaction is favored. *Rd*FucI-catalyzed isomerization favored the reverse reaction, producing l-fucose from l-fuculose with approximately 90% yield at 30 °C and pH 7.

### Effect of temperature and pH on the activity of *Rd*FucI and equilibrium

**Fig. 3:**
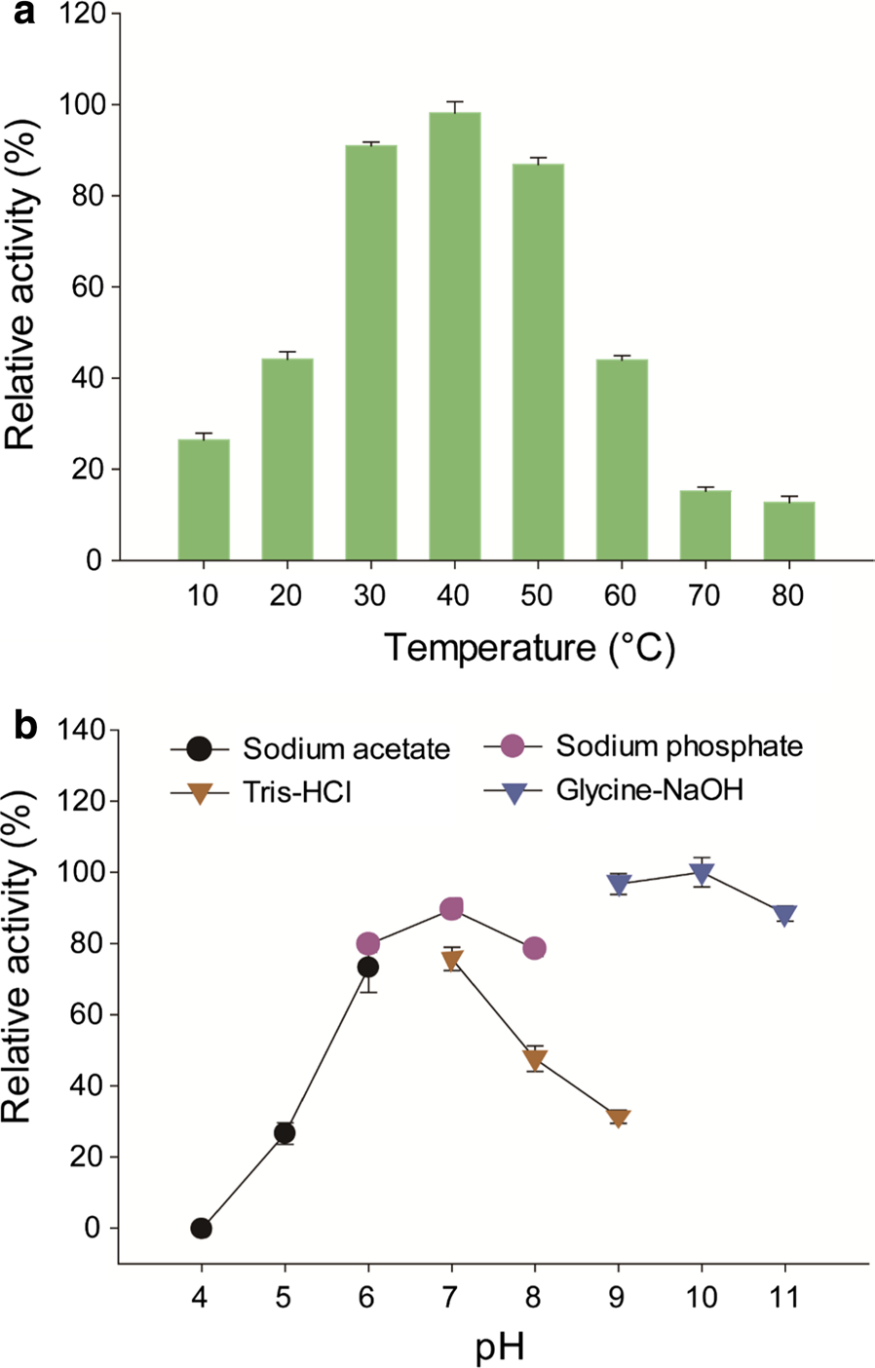
Effect of **a** temperature and **b** pH on the relative activity of *Rd*FucI against l-fuculose. Enzymatic reactions were performed **a** at various temperatures ranging from 10 to 80 °C and **b** at various pHs ranging from 4 to 11. The buffers used were 50 mM sodium acetate (pH 4, 5, and 6), 50 mM sodium phosphate (pH 6, 7, and 8), 50 mM Tris–HCl (pH 7, 8, and 9), and 50 mM glycine–NaOH (9, 10, and 11). Experimental data represent means ± standard deviations of three replicates

Enzymatic reactions were performed at various temperatures ranging from 10 to 80 °C and pHs ranging from 4 to 11 using l-fuculose as the substrate (Fig. 3). The isomerization of l-fuculose to l-fucose by *Rd*FucI was highly dependent on temperature, and maximal or near-maximal activities (> 80% of the maximum) were exhibited at temperatures ranging from 30 to 50 °C (Fig. 3a). To investigate the effect of temperature on the equilibrium for l-fucose to l-fuculose isomerization, the equilibrium ratio was investigated at 30, 40, and 50 °C at which maximal or near-maximal activities (> 80% of the maximum) were shown. As a result, there was no significant difference in equilibrium ratio among the three temperatures (l-fucose/l-fuculose = 9:1; *p* > 0.05). In other words, l-fucose was synthesized from l-fuculose with a yield of approximately 90% at all tested temperatures (Additional file 3: Fig. S3a).

The effect of pH was investigated. High activities of *Rd*FucI (> 70% of the maximum) were observed at alkaline and near neutral pHs (pHs 9, 10, and 11 and pHs 6, 7, and 8). Below pH 6, the enzyme activity decreased sharply, with little activity observed at pH 4. Despite the high specific activities at alkaline conditions, the l-fucose yield at equilibrium (60 min incubation) was much lower at pH 10 (54%) than at pH 7 (88%) (Additional file 3: Fig. S3b). The relative activities at pH 7, 8, and 9 were much lower in Tris–HCl buffer than the activities in sodium acetate or glycine–NaOH buffer, implying that Tris strongly inhibited the enzymatic activity of *Rd*FucI. The preceding enzymatic experiments in this study have been performed in reaction mixtures containing Tris at 1 mM, which was from the buffer change step after enzyme purification. To examine whether Tris in the reaction mixture could inhibit *Rd*FucI, isomerization activities from the reactions occurring in the absence and presence of 1 mM Tris were compared (Additional file 4: Fig. S4). No significant difference was evident in the enzymatic activities from the two reactions, indicating that 1 mM Tris did not inhibit *Rd*FucI activity.

### Effect of metal ions on the activity of *Rd*FucI

Sugar isomerases, including l-fucose isomerases, require divalent cations, such as Mn^2+^ and Co^2+^, as cofactors for their isomerization activities [14, 23, 24]. To study the effect of divalent cations on the catalytic activity of *Rd*FucI on l-fuculose, the enzyme activity was assayed in the absence and presence of either 1 mM of various metal ions or ethylenediaminetetraacetic acid (EDTA) (Table 1). The native *Rd*FucI enzyme not exposed to metal ions or EDTA displayed low activity, and metal chelation by EDTA reduced the enzyme activity. Among the tested metal ions, Mn^2+^, Mg^2+^, Co^2+^, Cd^2+^, and Zn^2+^ resulted in a pronounced increase in the enzyme activity. In particular, the addition of Mn^2+^ maximally enhanced activity of *Rd*FucI by approximately 7.4-fold. In contrast, Ca^2+^, Cu^2+^, and Fe^3+^ rather inhibited the activity of *Rd*FucI.

**Table 1:** Effect of metal ions on the activity of *Rd*FucI

Metal ion	Relative activity (%)^a^
Control^b^	100 ± 3
EDTA	77 ± 12
MnCl_2_	738 ± 70
MgCl_2_	533 ± 31
CoCl_2_	353 ± 83
CdCl_2_	183 ± 9
ZnCl_2_	159 ± 4
CsCl_2_	101 ± 16
LiSO_4_	101 ± 15
NiCl_2_	91 ± 13
FeCl_3_	74 ± 3
CaCl_2_	64 ± 8
CuCl_2_	58 ± 7

^a^Experimental data represent means ± standard deviations of three replicates

^b^Control represents the enzyme reaction not treated with EDTA or metal ions

### Substrate specificity and kinetic parameters of *Rd*FucI

**Fig. 4:**
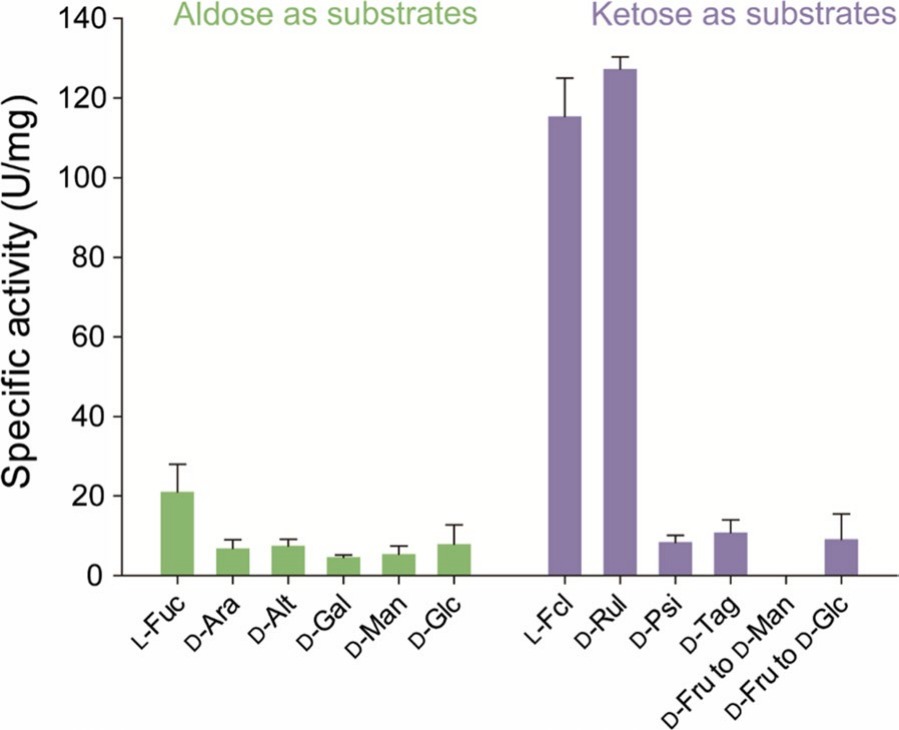
Substrate specificity of *Rd*FucI. Enzyme reactions were performed against 10 mM of various aldose and ketose substrates at 40 °C and pH 10. For aldose substrates, l-fucose, d-arabinose, d-altrose, d-galactose, d-mannose, and d-glucose were used. For ketose substrates, l-fuculose, d-ribulose, d-psicose, d-tagatose, and d-fructose were used. Experimental data represent means ± standard deviations of three replicates

In general, sugar and sugar phosphate isomerases display a broad specificity toward various substrates [14, 18, 19, 24]. To assess whether ketose-favoring activity of *Rd*FucI shown with l-fuculose was also evident with other substrates, the substrate specificity of *Rd*FucI was investigated against various aldose sugars (l-fucose, d-arabinose, d-altrose, d-galactose, d-mannose, and d-glucose) and their corresponding ketose sugars (l-fuculose, d-ribulose, d-psicose, d-tagatose, and d-fructose) (Fig. 4). Among all these substrates, including aldose and ketose sugars, the highest activities were observed with l-fuculose (115.3 U/mg) and d-ribulose (127.3 U/mg), which are both ketose sugars. The activities of *Rd*FucI for l-fuculose and d-ribulose were much higher than those for the other substrates. Among aldose sugars, the activity for l-fucose was the highest (21.0 U/mg), with the other substrates producing specific activities ranging from 4.7 to 7.9 U/mg. Ketose sugars other than l-fuculose and d-ribulose displayed specific activities from 0.0 to 10.8 U/mg. Thus, l-fuculose and d-ribulose were the preferred substrates for *Rd*FucI and ketose-favoring activity of *Rd*FucI was shown only with l-fuculose and d-ribulose.

Kinetic parameters were determined using l-fuculose and d-ribulose as the substrates (Additional file 5: Table S1). The values of *K*_m_ (Michaelis constant) and *k*_cat_ (turnover number of substrate) for l-fuculose were 1.9- and 1.2-fold lower, respectively, than those for d-ribulose. The catalytic efficiency of *Rd*FucI, represented as *k*_cat_*/K*_m_, for l-fuculose, was 1.5-fold higher than that of d-ribulose, indicating that l-fuculose is preferred as a substrate of *Rd*FucI.

### Overall crystal structure of *Rd*FucI

**Fig. 5:**
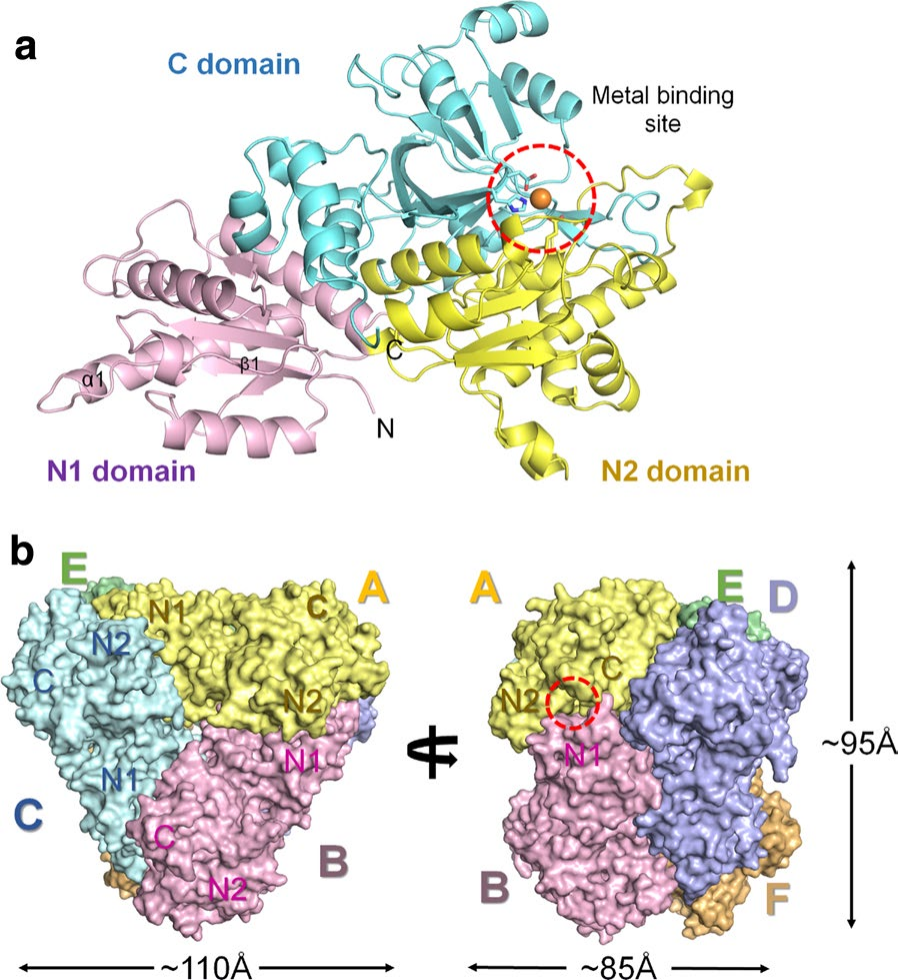
Overall structure of *Rd*FucI. **a** Cartoon representation of the *Rd*FucI monomer. *Rd*FucI monomer is composed of N1- (yellow), N2- (pink), and C-(green) domains. **b** Surface representation of the *Rd*FucI hexamer. Subunit A, B, C, D, E, and F are colored by yellow, pink, cyan, purple, green, and orange, respectively. A metal binding site on a substrate binding pocket site is indicated by a red dot

To better understand the molecular function, we determined the crystal structure of *Rd*FucI (Additional file 6: Table S2). The electron density map of *Rd*FucI was well defined from residues Ser5–Arg591 for six subunits in the asymmetric unit. The monomer *Rd*FucI consists of 19 α-helices and 23 β-strands comprising N1, N2, and C domains (Fig. 5a). The N1 domain (Ser5–Met172) adopts an α/β-fold and is involved in the substrate recognition of the hexameric formation of *Rd*FucI. N2 (Lys173–Leu352) and C (Thr353–Arg591) domains contain the metal binding residues involved in the catalytic activity (Fig. 5a). In the asymmetric unit, *Rd*FucI subunits form the hexameric formation arranged as a dimer of trimers with D_3_h pseudosymmetry (Fig. 5b). This is consistent with the result from analytical size-exclusion chromatography in which *Rd*FucI was revealed to exist as a homohexamer in solution (Additional file 7: Fig. S5).

In the hexameric formation, subunit A (total surface area: 23011.7 Å^2^) interacts with four different subunits B (residues in the interface: 47/buried surface area: 1909.6 Å^2^), C (58/1837.6 Å^2^), D (42/1482.9 Å^2^), and E (34/1086.2 Å^2^). Subunit A does not interact with the remaining subunit F (Fig. 5b). Subunit A of *Rd*FucI has a total buried surface area of 2569.1 Å^2^, representing 27.45% of the total surface area. This buried interface is stabilized by interaction involving 59 hydrogen bonds and 26 salt bridges from other subunits (Additional file 8: Table S3, Additional file 9: Table S4, Additional file 10: Table S5, Additional file 11: Table S6).

### Substrate binding site and active site of *Rd*FucI

**Fig. 6:**
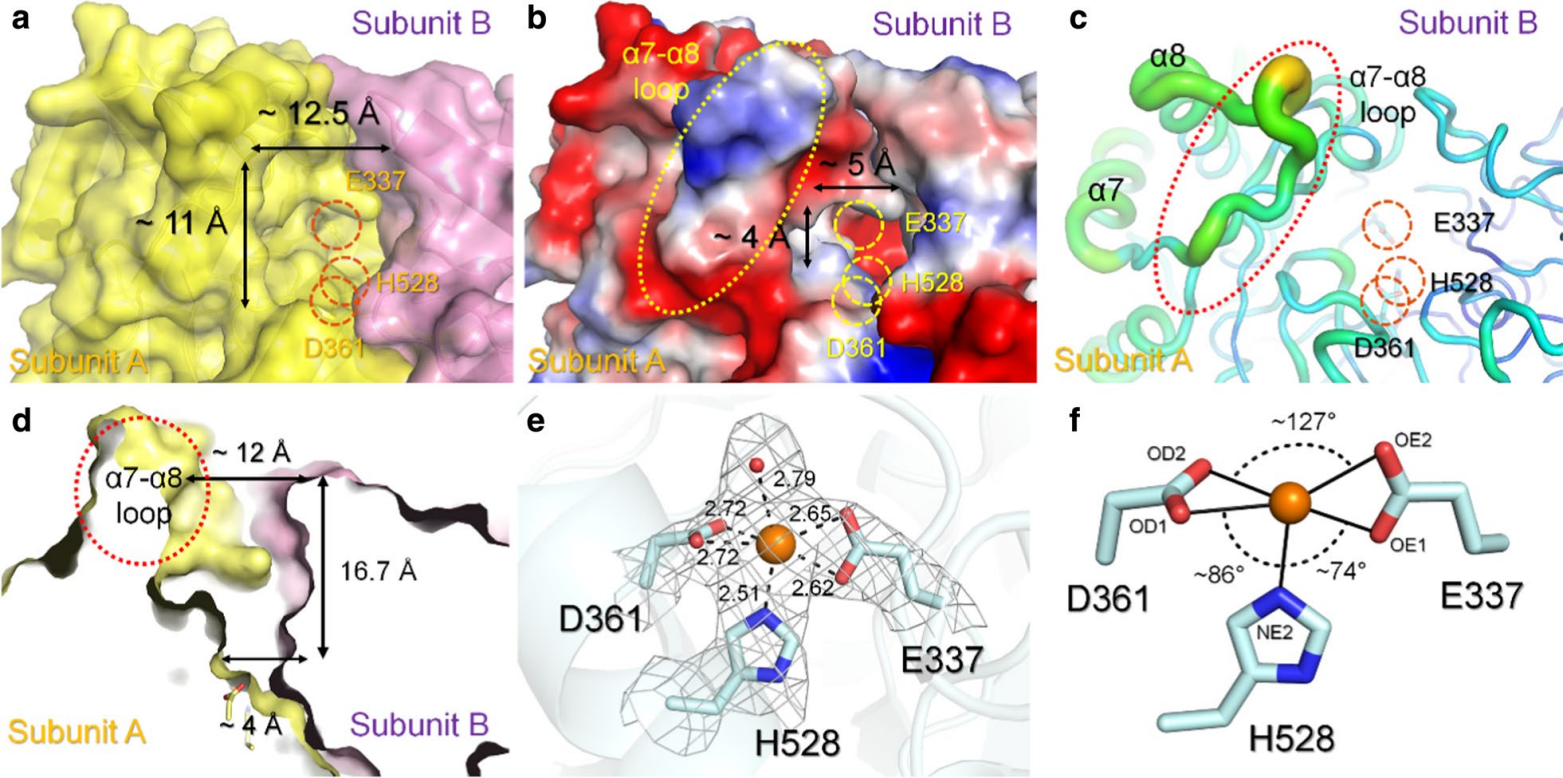
Substrate binding pocket and active site of *Rd*FucI. **a** The substrate binding pocket is formed by assembly by subunits A and C. **b** Electrostatic surface of substrate binding pocket. **c** B-factor presentation of the substrate binding surface. **d** Sectional surface view of the substrate biding pocket. **e** The 2Fo–F electron density map (gray mesh, contoured at 1.0 *σ*) on the metal binding sites of *Rd*FucI soaked into solution containing the 10 mM Mn^2+^. **f** Geometric analysis of the Mn^2+^ binding sites of *Rd*FucI

The substrate binding pocket is formed by the N2 and C domains of subunit A and the N1 domain of subunit B (Fig. 6a–c) and has a total of six substrate binding sites in the homohexameric *Rd*FucI. The entrance of the substrate binding pocket, where the substrate approaches, is approximately 11 × 12.5 Å (Fig. 6a). The substrate binding pocket, where the metal binding site is formed, has a negatively charged surface of approximately 4 × 5 Å (Fig. 6b). The distance between the metal binding site and the surface of the substrate binding pocket is approximately 16.7 Å (Fig. 6d), which implies that the active center is located deep in a pocket. This indicates that both the open chain and ring form of the substrate are accessible to the center of the active site and that, conversely, a bulk saccharide would not be accessible to the active site existing in the interior of the substrate binding pocket.

*Rd*FucI requires divalent metal ions for its catalytic activity for isomerization reaction using the ene-diol mechanism [23]. The metal binding site of *Rd*FucI should be coordinated with Mn^2+^ by conserved Glu337, Asp359, and His528 residues (Additional file 12: Fig. S6). However, there is no Fo-Fc electron density map (counted at > 5*σ*) that is suspected to be bound to Mn^2+^ as an essential metal for substrate binding (Additional file 13: Fig. S7a). The B-factor analysis revealed that the temperature factor of Mn^2+^ (70.53 Å^2^) is higher than the average temperature factor of the protein (36.22 Å^2^), indicating that Mn^2+^ is present on *Rd*FucI with a low occupancy. The finding was consistent with the result from the biochemical analysis in which the native enzyme displayed a low level of activity. On the other hand, the addition of Mn^2+^ substantially increased the catalytic activity of *Rd*FucI (Table 1). Thus, we speculated that the addition of Mn^2+^ to the *Rd*FucI crystal would increase the binding occupancy of Mn^2+^. After soaking *Rd*FucI crystals in a solution of 10 mM Mn^2+^, reliable Fo-Fc electron density (> 6*σ*) on the metal binding site was observed where the position of Mn^2+^ was clarified in all subunits (Fig. 6e and Additional file 13: Fig. S7b). However, the temperature B-factor of the Mn^2+^ (76.56 Å^2^) was higher than that of the whole protein (60.69 Å^2^), implying that Mn^2+^ ion is still not fully occupied in the metal binding site. Mn^2+^ was coordinated by OE1 (average distance: 2.62 Å) and OE2 (2.65 Å) atoms of Glu337, OD1 (2.72 Å) and OD2 (2.72 Å) atoms of Asp361, NE2 (2.52 Å) atom of His528, and the water molecule (2.79 Å) (Fig. 6e). The average bond angles of Glu337(OE1)–Mn^2+^–Asp361(OD2), Glu337(OE1)–Mn^2+^–His528(NE2), and Asp361(OD1)–Mn^2+^–His528(NE2) were 127.32°, 86.25°, and 73.96°, respectively. The bond angle between ligands and Mn^2+^ showed a distorted octahedral coordination.

### Structural comparison with other l-FucIs

**Fig. 7:**
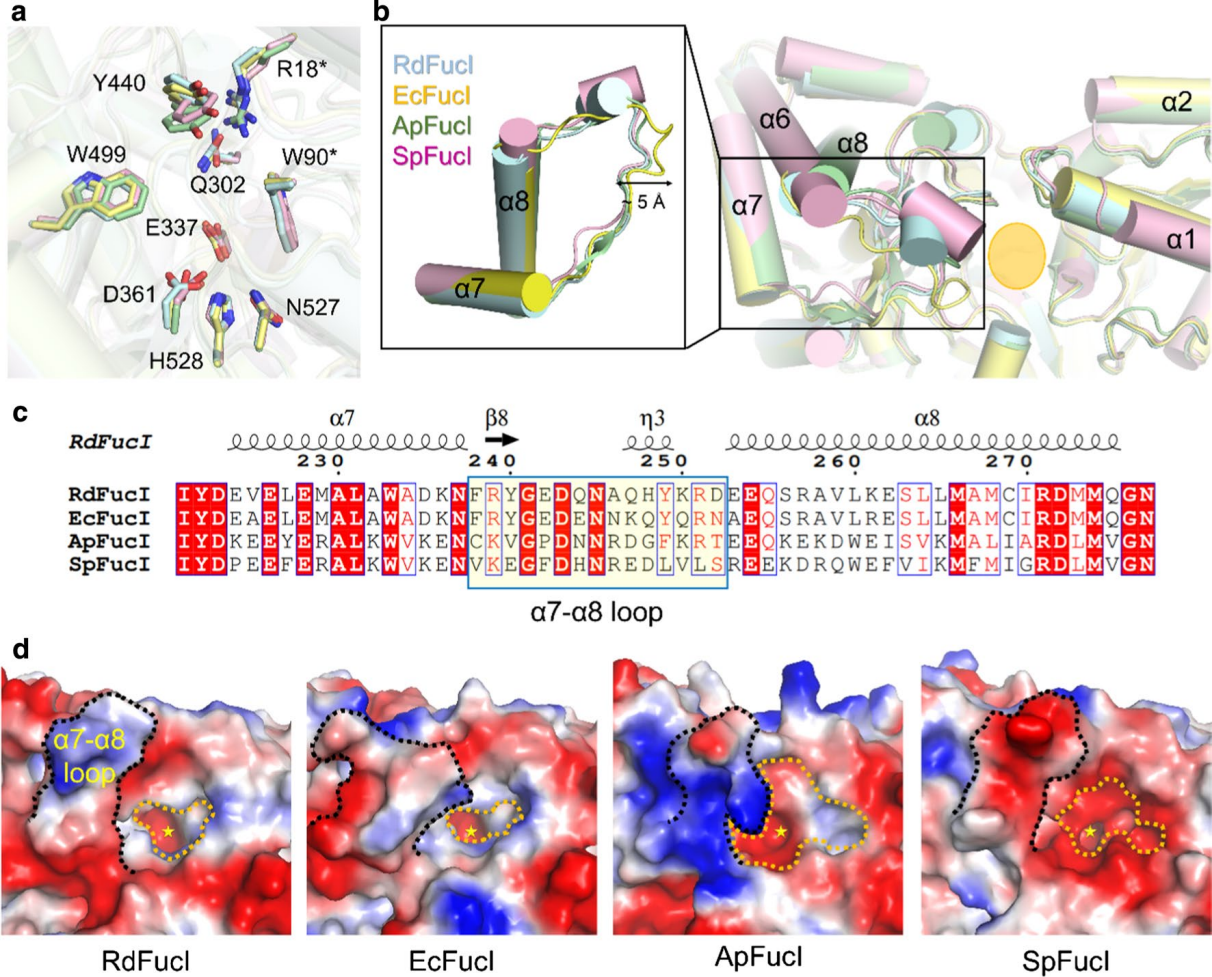
Structural comparison of *Rd*FucI with other l-FucIs. Superimposition of **a** active site and **b** substrate binding surface of *Rd*FucI with *Ec*FucI (PDB code: 1FUI), *Ap*FucI (3A9R) and *Sp*FucI (4C20). Close-up view of large conformational difference of α8–α9 loops from l-FucIs (left). **c** Partial sequence alignment for the α8–α9 loop region for the *Rd*FucI and *Ec*FucI, *Ap*FucI, and *Sp*FucI. **d** Electrostatic surface representation of *Rd*FucI, *Ec*FucI, *Ap*FucI, and *Sp*FucI. The deep substrate binding pocket and α8–α9 loop regions are indicated by orange- and black-dot lines, respectively. A metal binding site is indicated by a yellow asterisk

The DALI server was used to search for structural homologs. This search revealed that *Rd*FucI is similar to l-FucIs from *E. coli* (*Ec*FucI, PDB code 1FUI, *Z* score: 60.6, rmsd: 0.3 for 587 Cαs atoms), *Aeribacillus pallidus* (*Ap*FucI, 3A9R, *Z* score: 56.6, rmsd: 0.7 for 580 Cαs atoms), and *Streptococcus pneumonia* (*Sp*FucI, 4C20, *Z* score: 55.9, rmsd: 0.7 for 585 Cαs atoms). Superimposition of the substrate binding pocket showed that the metal binding residues Glu337, Asp361, and His528 (numbered in *Rd*FucI) are positionally identical to the other proteins, whereas substrate recognition residues (Arg16, W88, Gln300, Tyr437, Trp496, and Asn524) have a slight conformational difference in their side chains (Fig. 7a). In particular, the α7–α8 loop of each l-FucI, which lies on the surface of the substrate binding pocket, has a different conformation. Sequence alignment of l-FucIs showed a high similarity, but the sequence for α7–α8 loop of each l-FucI was highly variable (Fig. 7b). Since the α7–α8 loop is involved in forming the architecture of the substrate binding pocket, each l-FucI forms a unique substrate binding pocket (Fig. 7c). l-FucIs commonly have a negatively charged surface around the metal binding site, but the surface of the substrate binding pocket exhibits different charge states (Fig. 7c). As a result, the α7–α8 loop structural differences will cause differences in the substrate specificity of l-FucIs.

## Discussion

*Raoultella* sp. KDH14 isolated from abalone intestine is a novel species that possesses a gene cluster encoding putative l-fucose transporter (FucT), l-fucose mutarotase (FucU), l-fucose isomerase (FucI), l-fuculokinase (FucK), and l-fucose operon activator (FucR), indicating its potential involvement in l-fucose metabolism. Abalone feeds on brown seaweeds containing fucoidan and is a good source of fucoidan-degrading enzymes, which can degrade the polymeric fucoidan into its monomeric l-fucose [25–27]. In this study, *Raoultella* sp. KDH14 was isolated from an abalone intestine based on its ability to utilize fucoidan from *L. japonica*, in which the content of l-fucose was 34.1%, indicating that the strain potentially has fucoidan-degrading enzymes to generate l-fucose from fucoidan. This, along with the presence of putative genes for l-fucose metabolism, suggests that *Raoultella* sp. KDH14 is a good source for the study of l-FucI.

In the reversible reaction catalyzed by ketol isomerases, the strong formation of a certain sugar is not the general case. For example, when sweeteners d-fructose and d-tagatose are commercially produced by d-glucose and l-arabinose isomerases, respectively, a reactant and a product are present in a nearly equal equilibrium ratio (d-glucose/d-fructose = 6:4) [28] and (d-galactose/d-tagatose = 5.4:4.6) [29]. Accordingly, sugar synthesis using isomerase often encounters some difficulties with yield enhancement arising from equilibrium [28, 29]. The identified *Rd*FucI catalyzed the reverse reaction at a faster rate than it did the forward reaction, and equilibrium strongly favored the formation of the aldose l-fucose from the ketose l-fuculose at 30 °C and pH 7, as evidenced by the much higher portion of l-fucose in the reaction mixture at equilibrium (9:1). Therefore, the dominant reaction toward l-fucose that was shown with *Rd*FucI can be advantageous for industrial applications of l-fucose production. The equilibrium ratio between l-fucose and l-fuculose for *Rd*FucI was similar to those for previous *Ec*FucI (8.5:1.5) and *Kp*FucI (9:1) which catalyze the same reaction [16, 19] as the equilibrium is theoretically considered reaction dependent rather than enzyme independent.

In the cases of enzymatic isomerization of d-glucose to d-fructose and that of d-galactose to d-tagatose, the equilibrium is shifted by raising the reaction temperature [28, 30]. However, in this study, the equilibrium ratio between l-fucose and l-fuculose was not significantly altered by varying the temperature in the range of 30 to 50 °C, and thus the final yields of l-fucose from l-fuculose reached approximately 90% regardless of varying temperature. This may be because the tested temperatures were not different enough to shift the equilibrium ratio. Industrial processes often require a high temperature to prevent microbial contamination, to increase sugar solubility, and to minimize the viscosity of the reaction mixture [31]. In this study, both the relative specific activity of *Rd*FucI (reaction rate; 87% of the maximum) and the final yield of l-fucose (90%) still remain high at 50 °C, and were comparable to the enzymatic performance at 30 °C. Thus, *Rd*FucI could be applied for l-fucose synthesis at an elevated temperature, such as 50 °C. The l-fucose yield at equilibrium was much lower at pH 10. The reason could be the degradation of l-fucose and/or l-fuculose during long duration of incubation at the highly alkaline pH, rather than the equilibrium shift by pH [32]. This suggests that a highly alkaline pH condition is not desirable for the industrial production of l-fucose, as its final yield obtained at equilibrium was low regardless of the maximal specific activity.

From the pH profile, Tris was shown to act as an inhibitor of *Rd*FucI. The action of Tris and its analogue as the inhibitor has been already verified with other l-FucIs and sugar isomerases in which Tris inhibited sugar isomerases in non-competitive inhibition mode [28, 33]. Taken together, it is implied that the use of Tris, which is a widely used buffer for enzymatic reactions, should be avoided for the study or application of *Rd*FucI. For example, to avoid such chemical effect of using Tris–HCl buffer and to cover its pH range, the use of a mixture of sodium phosphate and glycine–NaOH could be an alternative. However, in our study, Tris at a concentration as low as 1 mM did not significantly inhibit the enzymatic activity of *Rd*FucI.

The isomerase activity of *Rd*FucI was maximized in the presence of Mn^2+^, whereas apo *Rd*FucI displayed much lower enzyme activity. The structural analysis of *Rd*FucI showed that apo *Rd*FucI contains Mn^2+^ with a low occupancy in the active site, whereas the occupancy of Mn^2+^ in the active site of *Rd*FucI was increased by the addition of Mn^2+^. The biochemical and structural results indicated that the active site of apo *Rd*FucI is not fully occupied by Mn^2+^ and, thus, isomerase activity was low. In contrast, the activity of isomerase activity was increased by addition of Mn^2+^ because the occupancy of Mn^2+^ was increased in the active site through additional Mn^2+^, which served as a platform for more substrate binding for isomerase activity. In general, Mn^2+^ prefers octahedral ligand geometry [34, 35]. Ideal bond angles between metal and ligand are stable at 90°, but acceptable ligand geometry exists between 30 and 120° [34, 35]. In *Rd*FucI, Mn^2+^ is coordinated by conserved Asp337, Glu361, and His528 residues, where the Mn^2+^ site has a distorted octahedral geometry of 73.96 to 127.32°. Therefore, the binding affinity of Mn^2+^ is considered to be low because *Rd*FucI does not stably coordinate the Mn^2+^ binding sites by the ligands. As a result, the addition of additional Mn^2+^ may increase the occupancy of this cation in the active site of *Rd*FucI.

l-FucIs reportedly catalyze the isomerization reaction of d-arabinose as well as l-fucose, in which the specific activities for l-fucose and d-arabinose were much higher than those for the other aldose substrates that were tested [16–18]. The behavior of FucIs that acts on both l-fucose and d-arabinose is closely related to the fact that l-FucI can be induced by either l-fucose or d-arabinose, and is involved in the metabolism of both l-fucose and d-arabinose [36, 37]. In this study, *Rd*FucI also catalyzed the interconversion of l-fucose and l-fuculose and that of d-arabinose and d-ribulose, although the reverse reaction was markedly favored. Such a strong preference of *Rd*FucI for l-fuculose and d-ribulose may arise from the identical configurations of hydroxyl groups at C3 and C4 positions of the two sugars. d-Arabinose, along with l-fucose, is also a rare sugar but of industrial significance due to its potential utilization as the starting material to synthesize antitumor compounds [38, 39]. Therefore, the dual substrate specificity of *Rd*FucI toward l-fuculose and d-ribulose will be helpful for the coproduction of commercially valuable l-fucose and d-arabinose sugars.

A comparison of the crystal structures of *Rd*FucI and other l-FucIs showed that the metal and substrate binding residues involved in the activity were positionally conserved. This indicates that *Rd*FucI would perform the equal isomerization reaction using the ene-diol mechanism that transfers the position of hydrogens from C2 to C1 and from O2 to O1 using Glu337 and Asp361, respectively, as previously reported with *Ec*FucI [23]. l-FucIs, on the other hand, have an α7–α8 loop at the entrance to the substrate binding pocket, which has a non-conserved sequence and a unique conformation. As a result, each l-FucI has its own pocket depth and width, which is considered to potentially affect its substrate binding and catalytic activity.

## Conclusions

We have performed biochemical and structural analyses of *Rd*FucI from the novel species of *Raoultella* isolated in our laboratory. This is the first study of an l-FucI from the *Raoultella* genus. The characteristic of *Rd*FucI that catalyzes dominant formation of aldose in interconversion of l-fucose to l-fuculose and that of d-arabinose to d-ribulose will be helpful for understanding of molecular function of l-FucIs as well as bacterial metabolism of l-fucose and d-arabinose. Furthermore, these results will facilitate developing the enzymatic synthesis of l-fucose and d-arabinose for industrial applications.

## Methods

### Gene cloning and expression of *Rd*FucI

The genomic DNA of *Raoultella* sp. strain KDH14 was used as the template for the amplification of a gene encoding a putative l-FucI (Accession No. MK893986) by polymerase chain reaction. Primers were designed to incorporate the *Nde*I and *EcoR*I restriction sites as follows: forward primer, 5′-G GAA TTC CAT ATG AAA AGA ATC AGC TTA CCA AAA ATT-3′ with NdeI site plus overhang (underlined); and a reverse primer, 5′-CG GAA TTC TTA ACG TTT ATA CAG CGG GCC-3′ with EcoRI site plus overhang (underlined). The amplified gene for *Rd*FucI was then ligated into the pET28a vector (Novagen, Darmstadt, Germany).

*Escherichia coli* BL21(DE3) was used for enzyme expression. An overnight culture of recombinant *E. coli* (20 ml) was inoculated into LB broth containing 50 µg/ml kanamycin (1000 ml) and cultivated at 37 °C with shaking at 180 rpm. When the cells reached an optical density of 0.6 to 0.8 at 600 nm, the expression of *Rd*FucI was induced by supplementing with 0.5 mM isopropyl-β-d-1-thiogalactopyranoside (IPTG), and the culture was incubated for an additional 16 h at 18 °C.

### Purification of *Rd*FucI

The cells harvested by centrifugation were resuspended in a buffer composed of 50 mM Tris–HCl and 200 mM NaCl with 20 mM imidazole (pH 8.0) (Buffer A) and then disrupted by sonication. The cell lysate obtained by centrifugation at 25,188×*g* and 4 °C for 30 min was applied to a His-Trap column (GE Healthcare, Chicago, IL) equilibrated with Buffer A. The recombinant *Rd*FucI was eluted with a buffer composed of 50 mM Tris–HCl (pH 8.0) and 200 mM NaCl with 300 mM imidazole (Buffer B). The eluted fractions were concentrated against a buffer containing 10 mM Tris–HCl and 200 mM NaCl using a centrifugal filter unit with a cutoff size of 30 kDa (Millipore, Burlington, MA) at 3500 1240×*g* at 4 °C. The fractions were then stored in a deep freezer (− 80 °C) until required.

### Size-exclusion chromatography

For crystallization and molecular mass analysis of the native *Rd*FucI protein, the concentrated *Rd*FucI was subjected to size-exclusion chromatography using a Superdex 200 10/300 GL column (GE Healthcare) equilibrated with a buffer consisting of 10 mM Tris–HCl and 200 mM NaCl (pH 8.0). The column was calibrated with standard proteins that included thyroglobulin (669 kDa), ferritin (440 kDa), bovine serum albumin (67 kDa), β-lactoglobulin (35 kDa), and ribonuclease A (13.7 kDa).

### Enzyme assay

Unless otherwise specified, the enzymatic reaction was performed using 1.5 µg of *Rd*FucI and 10 mM of l-fucose or l-fuculose as the substrate contained in a 20 mM sodium phosphate (pH 7) or 50 mM glycine–NaOH (pH 10) in the presence of 1 mM MnCl_2_ in a total volume of 100 µl. The enzyme reaction was terminated by boiling the reaction sample at 95 °C for 10 min. For time-course experiments, only l-fucose was assayed using the K-FUCOSE assay kit (Bray, Co. Wicklow, Ireland), according to the manufacturer’s instructions. Since this enzymatic reaction was conversion between l-fucose and l-fuculose, the amount of l-fuculose was determined by measuring the decreased amount of l-fucose by the enzymatic reaction.

To investigate the effect of temperature and pH, enzymatic reactions were performed at various temperatures ranging from 10 to 80 °C and pH 7.0, and at various pHs ranging from 4 to 11 using four buffer systems at 40 °C: 50 mM sodium acetate for pH 4 to 6, 50 mM sodium phosphate for pH 6 to 8, 50 mM Tris–HCl for pH 7 to 9, and 50 mM glycine–NaOH for pH 9 to 11. To examine the effect of metal ions, the enzyme activity was assayed in the presence of EDTA or various metal ions at 1 mM, including MnCl_2_, CaCl_2_ CuCl_2_, CdCl_2_, CoCl_2_, CsCl_2_, MgCl_2_, NiCl_2_, ZnCl_2_, FeCl_3_, and LiSO_4_. One unit (U) was defined as the amount of enzyme required to produce 1 µmol of aldose or ketose sugars per min.

### Substrate specificity

Various aldose sugars (l-fucose, d-arabinose, d-altrose, d-galactose, d-mannose, and d-glucose) and ketose sugars (l-fuculose, d-ribulose, d-psicose, d-tagatose, and d-fructose) were used for the enzymatic reaction performed at 40 °C for 5 min in which 1.5 µg *Rd*FucI was added to 50 mM glycine–NaOH (pH 10) containing 1 mM MnCl_2_. The amounts of aldose sugars converted from ketose sugars were determined by high-performance liquid chromatography system equipped with a refractive index detector and an SP0810 column (Bio-Rad Laboratories, Hercules, CA). Filtrated and degassed distilled water was used as the mobile phase, which was applied to the column set at 78 °C with a flow rate of 0.5 ml/min. The amounts of ketose sugars converted from aldose sugars were measured spectrophotometrically [40]. One hundred microliters of 1.5% (w/v) cysteine hydrochloride, 3 ml of 70% (v/v) H_2_SO_4_, and 100 µl of 0.12% (w/v) carbazole in absolute ethanol were successively added to the 100 µl reaction mixture, which was then incubated at 35 °C for 10 min. The absorbance was measured at 540 nm, and the amounts of sugars formed were calculated by the calibration curve using each ketose sugar as the standard.

### Protein crystallization

The initial crystallization screening of *Rd*FucI (30 mg/ml) was performed with the Index HT, Salt RX HT, and Crystal Screen HT commercially available kits (Hampton Research, Aliso Viejo, CA) using the sitting-drop vapor-diffusion method at 20 °C. Microcrystals were obtained by precipitation in a solution containing 0.1 M HEPES, pH 7.5, and 20% (w/v) polyethylene glycol 10,000. Suitable crystals for X-ray diffraction were obtained using the diluted *Rd*FucI (15 mg/ml) solution with the crystallization solution using the hanging-drop vapor-diffusion method at 20 °C.

### X-ray diffraction data collection from protein crystals

Crystals were soaked in a reservoir solution containing an additional 20% (v/v) glycerol, and flash-cooled in a nitrogen stream. X-ray diffraction datasets for the crystals were collected at 100 K on the beamline 11C at PLS-II (Pohang, Republic of Korea) using a Pilatus 6 M or on the beamline 6A using an ADSC Quantum Q270 CCD detector [41]. The diffraction data were processed using the HKL2000 program [42].

### Protein crystal structure determination and analysis

The phases were resolved using the molecular replacement method as implemented in MOLREP [43] using the crystal structure of *Ec*FucI (PDB code: 1FUI) [23] as the search model. The structure was manually rebuilt and refined using COOT [44]. The structural refinement was performed using REFMAC5 [45]. The structure quality was validated using MolProbity [46]. The refinement statistics are summarized in Additional file 5: Table S2. The final coordinates and structural factors have been deposited within the Protein Data Bank (PDB) under the accession codes 6K1F (*Rd*FucI) and 6K1G (*Rd*FucI soaked with Mn^2+^).

Interface areas between the subunits were calculated with PDBePISA. The structure-based sequence alignment was carried out using Clustal Omega [47] and ESPRIPT [48]. Structural homolog was searched using the DALI server [49]. Figures of the structure were prepared using PyMOL (https://pymol.org/).

## Supplementary information

**Additional file 1: Method.** Isolation and Identification of *Raoultella* sp. KDH14**. Fig. S1.** Phylogenetic position of *Raoultella* sp. KDH14 based on the 16S rRNA sequence.

**Additional file 2: Fig. S2.** TLC and GC/MS analyses for the identification of products synthesized by *Rd*FucI.

**Additional file 3: Fig. S3.** Effect of temperature and pH on l-fucose yield at equilibrium.

**Additional file 4: Fig. S4.** Effect of Tris on the enzymatic activity of *Rd*FucI.

**Additional file 5: Table S1.** Kinetic parameters of *Rd*FucI.

**Additional file 6: Table S2.** Data collection and refinement statistics for *Rd*FucI.

**Additional file 7: Fig. S5.** Analytical gel filtration chromatography profile of *Rd*FucI.

**Additional file 8: Table S3.** Hydrogen bonds and salt bridges on the A–B interface of *Rd*FucI.

**Additional file 9: Table S4.** Hydrogen bonds and salt bridges on the A–C interface of *Rd*FucI.

**Additional file 10: Table S5.** Hydrogen bonds and salt bridges on the A–D interface of *Rd*FucI.

**Additional file 11: Table S6.** Hydrogen bonds and salt bridges on the A–E interface of *Rd*FucI.

**Additional file 12: Fig. S6.** Structure-based sequence alignment of *Rd*FucI, *Ec*FucI, *Ap*FucI, and *Sp*FucI.

**Additional file 13: Fig. S7.** 2Fo-Fc and Fo-Fc electron density maps of metal binding site for (a) *Rd*FucI and (b) *Rd*FucI-Mn^2+^.

## Data Availability

All data generated or analyzed during this study are included in the published article and its additional files. DNA sequences and resequencing results are available from GenBank via their accession numbers.

## Abbreviations

l-FucI: l-Fucose isomerase
NCBI: National Center for Biotechnology Information
EDTA: ethylenediaminetetraacetic acid
PDB: Protein Data Bank
