# Supplementary material for: Enzymatic synthesis of l-fucose from l-fuculose using a fucose isomerase from Raoultella sp. and the biochemical and structural analyses of the enzyme
Source: Biotechnol Biofuels. 2019 Dec 5;12:282. doi: 10.1186/s13068-019-1619-0 (PMC6894278; doi:10.1186/s13068-019-1619-0)
Supplement: Supplementary file 1 — Additional file 1: Method. Isolation and Identification of Raoultella sp. KDH14. Fig. S1. Phylogenetic position of Raoultella sp. KDH14 based on the 16S rRNA sequence. [file 13068_2019_1619_MOESM1_ESM.docx]

**Additional file 1**

**Method** Isolation and Identification of *Raoultella* sp. KDH14

*Raoultella* sp. KDH14 was isolated from the intestine of abalone collected from an abalone breeding and production farm located at Yeosu-si, Jeollanam-do, Korea. The intestine was dissected and diluted using sterile water, followed by inoculation into M9 minimal medium containing 2% (w/v) fucoidan from *Laminaria japonica* (Sigma-Aldrich, St Louis, MO) and 2% (w/v) peptone (Sigma-Aldrich). The cells in the intestine were incubated at 30℃ for 3 days on a shaking incubator and then, 200 μl of each culture was spread on agar plates containing 2% (w/v) peptone, 1.5% (w/v) agar, and 2% (w/v) fucoidan as the sole carbon source. To examine the fucoidan-utilizing ability, 192 single colonies were isolated from agar plates and inoculated into the same medium as described above. Growth of individual colonies was measured using a Synergy HTX multi-mode reader (BioTek Instruments, Inc., Winooski, VT).

The genomic DNA of the isolated KDH14 strain was extracted using a commercial DNA isolation kit (Qiagen, Valencia, CA). The 16S rRNA sequence of isolated strain KDH14 was amplified by PCR using bacterial 16S rRNA primers 27F (5′-AGAGTTTGATCCTGGCTCAG-3′) and 1492R (5′-GGCTACCTTGTTACGACTT-3′). The resulting sequence was compared for similarities to RNA sequences in the NCBI database. The phylogenetic tree was constructed using MEGA 7.0 software using the maximum-likelihood (ML) and neighbor-joining (NJ) methods to examine the phylogenetic positions of the isolated strain KDH14 [1].

To perform complete genome sequencing of the strain KDH14, a total of 5 μg for each sample was used as input into the library preparation. The SMRTbell library was constructed with SMRTbell™ Template Prep Kit 1.0 (PN 100-259-100) following the manufacturer’s instructions (Pacific Biosciences, Menlo Park, CA). De novo assembly was conducted using the hierarchical genome assembly process (HGAP, Version 2.3) workflow [2], including consensus polishing with Quiver. Putative gene coding sequences (CDSs) from the assembled contigs were identified using Glimmer v3.02 [3] and open reading frames (ORFs). These ORFs were searched using Blastall alignment against the NCBI Non-redundant protein database (nr) for all species. GO annotation was assigned to each of ORFs by Blast2GO software [4], analyzing the best hits of the BLAST results.

**
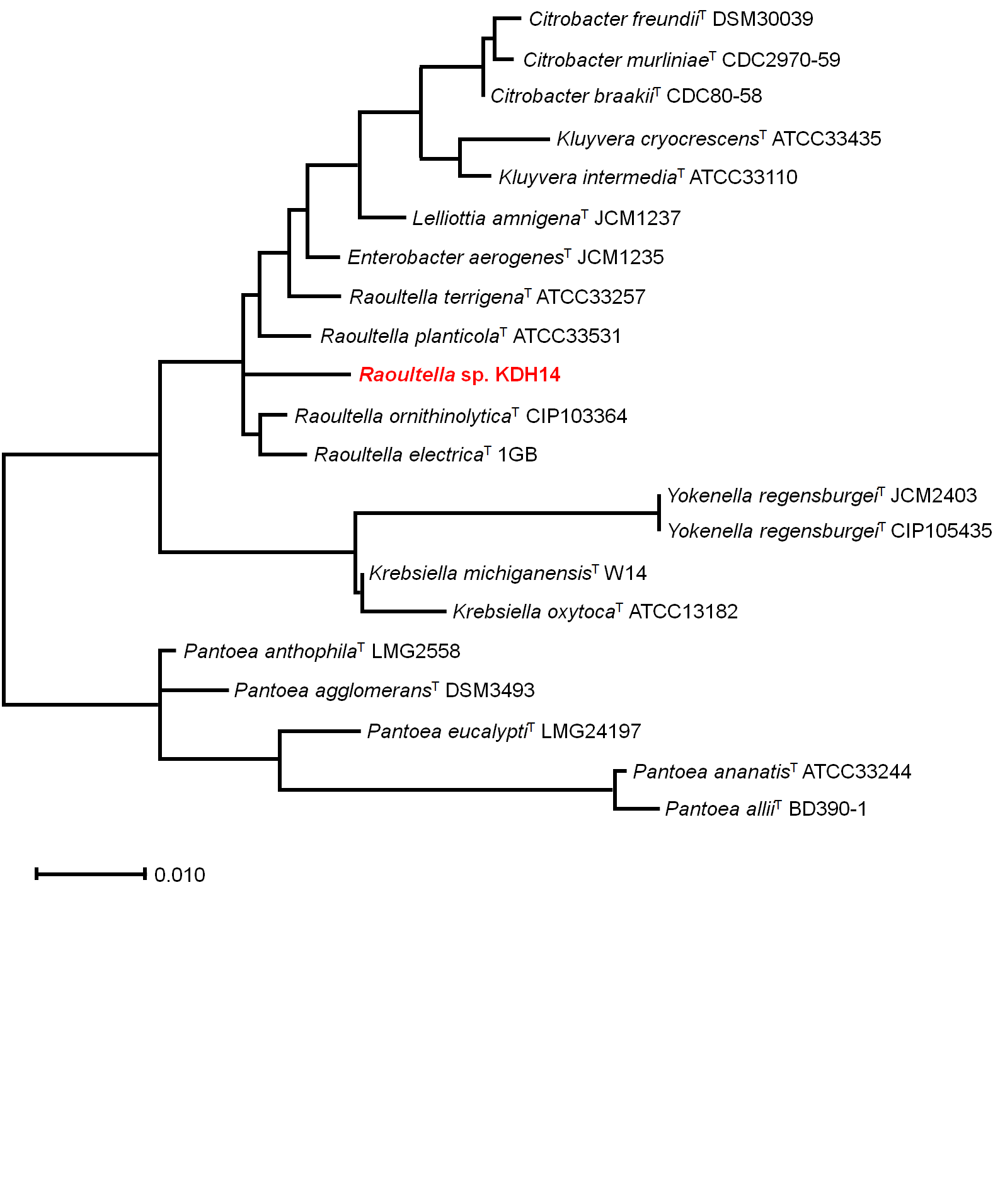
**

**Fig. S1** Phylogenetic position of *Raoultella* sp. KDH14 based on the 16S rRNA sequence analysis
