# Supplementary material for: Enzymatic synthesis of l-fucose from l-fuculose using a fucose isomerase from Raoultella sp. and the biochemical and structural analyses of the enzyme
Source: Biotechnol Biofuels. 2019 Dec 5;12:282. doi: 10.1186/s13068-019-1619-0 (PMC6894278; doi:10.1186/s13068-019-1619-0)
Supplement: Supplementary file 2 — Additional file 2: Fig. S2. TLC and GC/MS analyses for the identification of products synthesized by RdFucI. [file 13068_2019_1619_MOESM2_ESM.docx]

**Additional file 2**


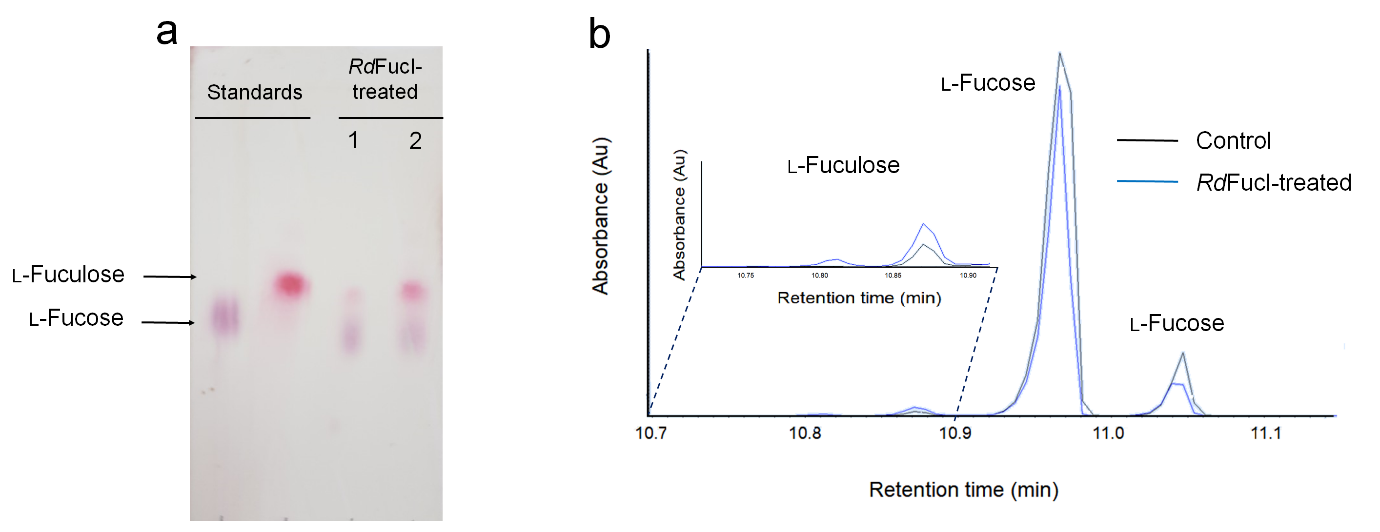


**Fig. S2** TLC and GC/MS analyses for the identification of products synthesized by *Rd*FucI. (a) TLC analysis for the identification of products from forward reaction (Lane 1: l-fucose to l-fuculose) and from reverse reaction (Lane 2: l-fuculose to l-fucose). (b) GC/MS analysis for the identification of l-fuculose synthesized from l-fucose by *Rd*FucI. The inset is the expanded chromatogram for l-fuculose peak
