# Supplementary material for: Enzymatic synthesis of l-fucose from l-fuculose using a fucose isomerase from Raoultella sp. and the biochemical and structural analyses of the enzyme
Source: Biotechnol Biofuels. 2019 Dec 5;12:282. doi: 10.1186/s13068-019-1619-0 (PMC6894278; doi:10.1186/s13068-019-1619-0)
Supplement: Supplementary file 3 — Additional file 3: Fig. S3. Effect of temperature and pH on l-fucose yield at equilibrium. [file 13068_2019_1619_MOESM3_ESM.docx]

**Additional file 3**

**
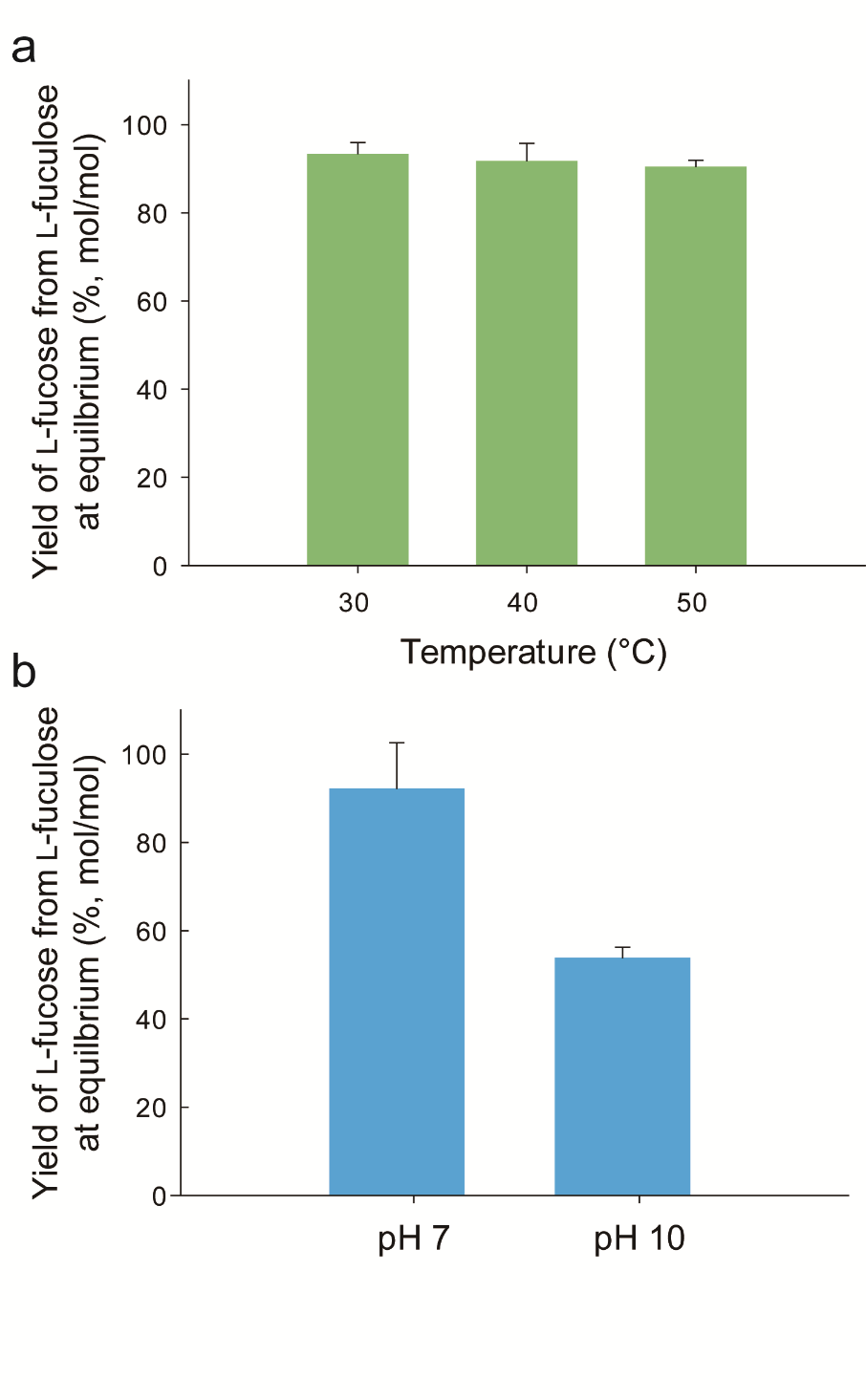
**

**Fig. S3** Effect of temperature and pH on l-fucose yield at equilibrium. (a) The enzymatic reaction was performed at 30, 40, and 50°C in 20 mM sodium phosphate buffer (pH 7) for 60 min. (b) The enzymatic reaction was performed at 40°C in 50 mM glycine-NaOH buffer (pH 10) for 60 min. Both reactions used 10 mM l-fuculose as the substrate. Yield of l-fucose (%, mol/mol) represents the percentage ratio of the molar amount of l-fucose converted from l-fuculose to the theoretical yield
