# Supplementary material for: Enzymatic synthesis of l-fucose from l-fuculose using a fucose isomerase from Raoultella sp. and the biochemical and structural analyses of the enzyme
Source: Biotechnol Biofuels. 2019 Dec 5;12:282. doi: 10.1186/s13068-019-1619-0 (PMC6894278; doi:10.1186/s13068-019-1619-0)
Supplement: Supplementary file 4 — Additional file 4: Fig. S4. Effect of Tris on the enzymatic activity of RdFucI. [file 13068_2019_1619_MOESM4_ESM.docx]

**Additional file 4**


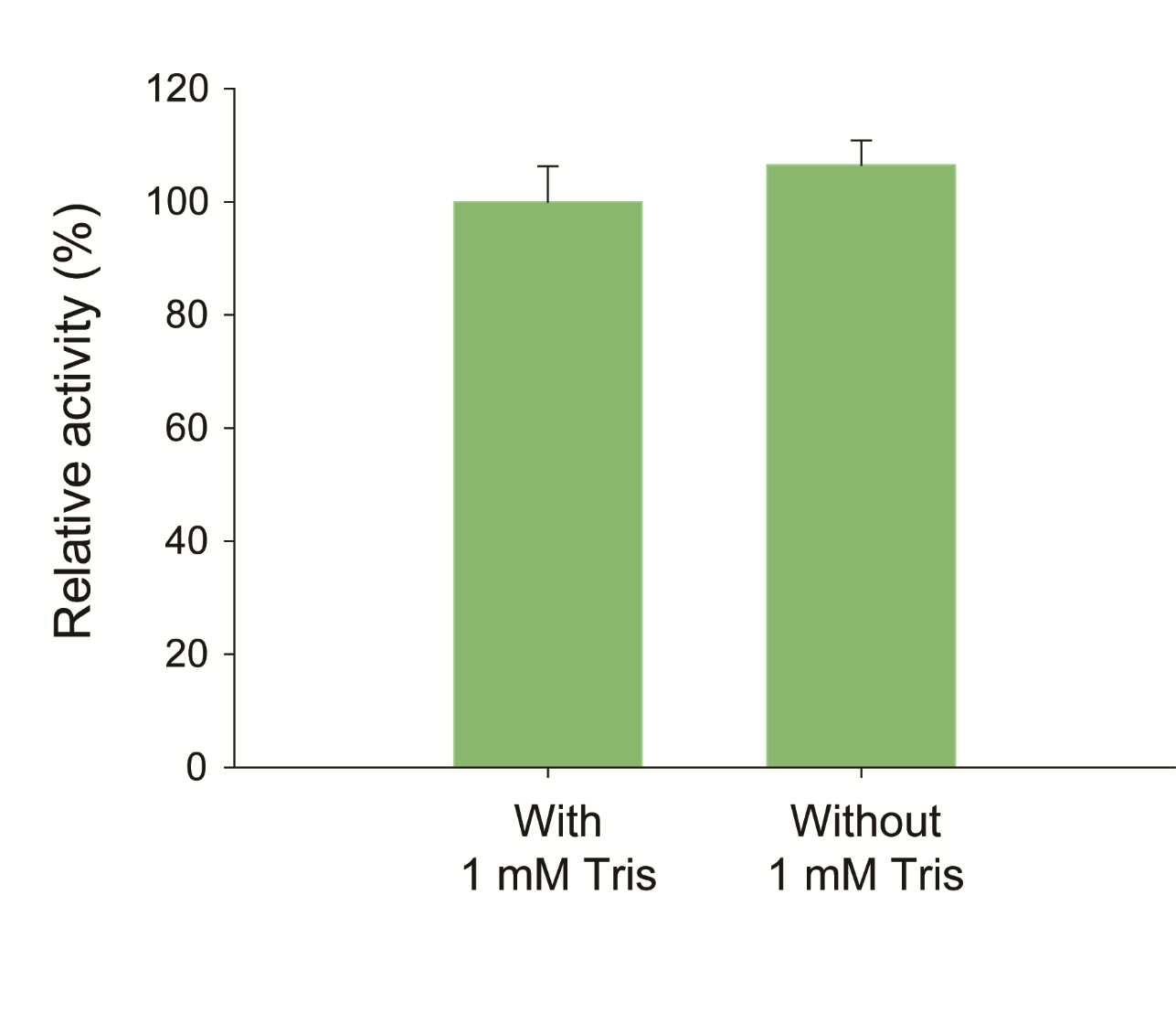


**Fig. S4** Effect of Tris on the enzymatic activity of *Rd*FucI. The enzymatic reaction was performed in 50 mM glycine-NaOH (pH 10) containing 10 mM l-fuculose, 1.5 µg *Rd*FucI, and 1 mM MnCl_2_ in the presence or absence of 1 mM Tris at 40°C for 5 min. The addition of 1 mM Tris did not significantly change the pH of the reaction mixture
