# Supplementary material for: Enzymatic synthesis of l-fucose from l-fuculose using a fucose isomerase from Raoultella sp. and the biochemical and structural analyses of the enzyme
Source: Biotechnol Biofuels. 2019 Dec 5;12:282. doi: 10.1186/s13068-019-1619-0 (PMC6894278; doi:10.1186/s13068-019-1619-0)
Supplement: Supplementary file 5 — Additional file 5: Table S1. Kinetic parameters of RdFucI. [file 13068_2019_1619_MOESM5_ESM.docx]

**Additional file 5**

**Table S1** Kinetic parameters of *Rd*FucI^a,b^

| Substrate | Product | *K_m_*  (mM) | *k_cat_*  (min^-1^) | *k_cat/_K_m_*  (mM^- 1^ min^-1^) |
| --- | --- | --- | --- | --- |
| l-Fuculose | l-Fucose | 7.3 ± 0.2 | 6805.5 ± 11.3 | 928.3 ± 29.1 |
| d-Ribulose | d-Arabinose | 13.7 ± 1.1 | 8278.8 ± 341.5 | 608.0 ± 29.6 |

^a^Various concentrations of l-fuculose and d-ribulose ranging from 0.625 to 50 mM were incubated with 1.5 µg *Rd*FucI in 50 mM glycine-NaOH (pH 10) at 40ºC for 5 min

^b^Experimental data represent means ± standard deviations of three replicates
