# Supplementary material for: Enzymatic synthesis of l-fucose from l-fuculose using a fucose isomerase from Raoultella sp. and the biochemical and structural analyses of the enzyme
Source: Biotechnol Biofuels. 2019 Dec 5;12:282. doi: 10.1186/s13068-019-1619-0 (PMC6894278; doi:10.1186/s13068-019-1619-0)
Supplement: Supplementary file 6 — Additional file 6: Table S2. Data collection and refinement statistics for RdFucI. [file 13068_2019_1619_MOESM6_ESM.docx]

**Additional file 6**

**Table S2** Data collection and refinement statistics for *Rd*FucI

| **Data collection** | *Rd*FucI | *Rd*FucI-Mn^2+^ |
| --- | --- | --- |
| Space group | P2_1_2_1_2_1_ | P2_1_2_1_2_1_ |
| Cell dimensions |  |  |
| *a*, *b*, *c* (Å) | 113.98, 127.61, 257.29 | 116.27, 163.27, 196.34 |
| Resolution (Å) | 50.0–2.50 (2.54–2.50)^a^ | 30.0-2.95 (3.00–2.95)^a^ |
| Completeness | 96.4 (95.4)^a^ | 93.8 (85.9)^a^ |
| Redundancy | 4.1 (3.2)^a^ | 4.4. (3.3)^a^ |
| I/σ(I) | 7.65 (1.59)^a^ | 17.34 (2.29)^a^ |
| R_merge_^b^ | 0.115 (0.357)^a^ | 0.120 (0.463)^a^ |
| **Refinement statistics** |  |  |
| Resolution (Å) | 50.0–2.50 | 30.0–2.96 |
| R_work_ / R_free_ (%)^c^ | 17.65/23.33 | 19.26/25.19 |
| B-factor (Averaged) |  |  |
| Protein | 36.22 | 60.69 |
| Water | 29.25 | 38.77 |
| R.M.S. deviations |  |  |
| Bond lengths (Å) | 0.011 | 0.010 |
| Bond angles (°) | 1.394 | 1.320 |
| Ramachandran plot (%) |  |  |
| favored | 95.9 | 95.1 |
| allowed | 3.7 | 4.3 |
| Outliers | 0.4 | 0.6 |

^a^The highest resolution shell is shown in parentheses

^b^*R*_merge_ = Σ*_h_*Σ*_i_*|I*i*(hkl)_<*I*(hkl)>|/Σ*_h_*Σ*_i_*I*_i_*(hkl), where *I_i_*(hkl) is the intensity of the ‘ith’ measurement of reflection hkl and <*I*(hkl)> is the weighted mean of all measurements of hkl

^c^*R*_work_ = Σ||*F*_obs_|-|*F*_calc_||/Σ|*F*_obs_|, where *F*_obs_ and *F*_calc_ are the observed and calculated structure-factor amplitudes, respectively. R_free_ was calculated as R_work_ using a randomly selected subset (5.1%) of unique reflections not used for structure refinement
