# Supplementary material for: Enzymatic synthesis of l-fucose from l-fuculose using a fucose isomerase from Raoultella sp. and the biochemical and structural analyses of the enzyme
Source: Biotechnol Biofuels. 2019 Dec 5;12:282. doi: 10.1186/s13068-019-1619-0 (PMC6894278; doi:10.1186/s13068-019-1619-0)
Supplement: Supplementary file 7 — Additional file 7: Fig. S5. Analytical gel filtration chromatography profile of RdFucI. [file 13068_2019_1619_MOESM7_ESM.docx]

**Additional file 7**


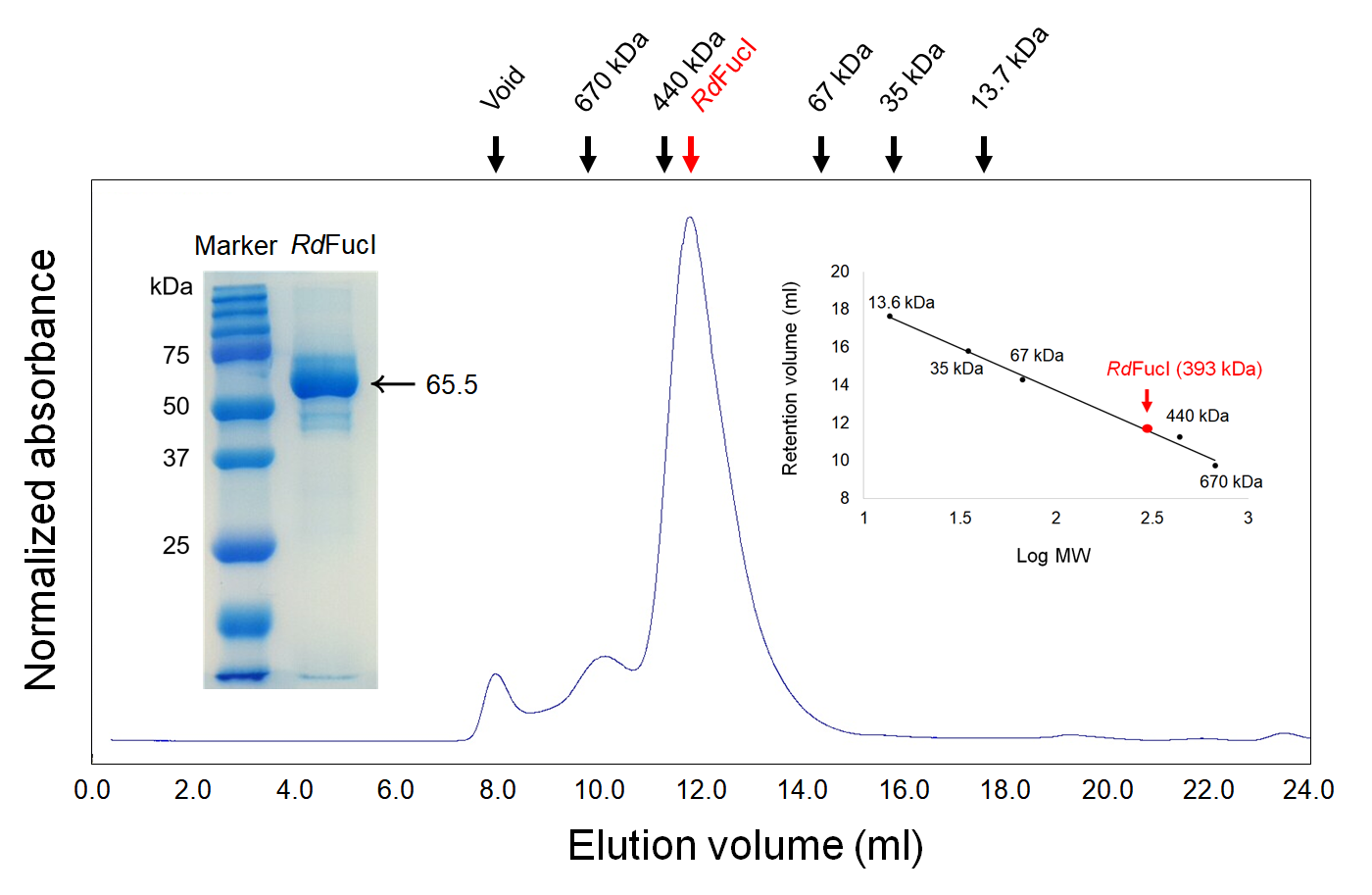


**Fig. S5** Analytical gel filtration chromatography profile of *Rd*FucI. The molecular weights of the protein standards are indicated by arrows at the top. The picture on the left represents SDS-PAGE gel analysis of *Rd*FucI purified by affinity chromatography using the His-Trap column
