# Supplementary material for: Enzymatic synthesis of l-fucose from l-fuculose using a fucose isomerase from Raoultella sp. and the biochemical and structural analyses of the enzyme
Source: Biotechnol Biofuels. 2019 Dec 5;12:282. doi: 10.1186/s13068-019-1619-0 (PMC6894278; doi:10.1186/s13068-019-1619-0)
Supplement: Supplementary file 8 — Additional file 8: Table S3. Hydrogen bonds and salt bridges on the A–B interface of RdFucI. [file 13068_2019_1619_MOESM8_ESM.docx]

**Additional file 8**

**Table S3** Hydrogen bonds and salt bridges on the A-B interface of *Rd*FucI

| **Chain A**  **(residue [atom])** | **Distribution [Å]** | **Chain B**  **(residue [atom])** |
| --- | --- | --- |
| Tyr92 [OH] | 2.9 | Arg303 [NH1] |
| Thr113 [O] | 2.8 | Arg363 [NH1] |
| Thr113 [OG1] | 3.4 | Arg494 [NH1] |
| Ser129 [O] | 3.1 | Lys467 [N] |
| Tyr139 [O] | 2.6 | Tyr518 [OH] |
| Asp142 [OD1] | 2.5 | Ser367 [OG] |
| Val143 [O] | 2.7 | Arg373 [NH1] |
| Gln144 [O] | 3.7 | Arg494 [NH1] |
| Ala569 [O] | 2.8 | Tyr579 [OH] |
| Arg18 [NH1] | 3.0 | Tyr440 [O] |
| Arg18 [NH2] | 2.7 | Gln302 [OE1] |
| Gly93 [N] | 3.0 | Met185 [O] |
| Ser94 [N] | 2.8 | Gly186 [O] |
| Ser94 [OG] | 3.2 | Gly186 [O] |
| Thr113 [OG1] | 2.4 | Arg494 [O] |
| Glu114 [N] | 3.5 | Arg494 [O] |
| Lys158 [NZ] | 3.0 | Tyr518 [OH] |
| Gly571 [N] | 3.0 | Tyr579 [OH] |
| Met572 [N] | 3.7 | Cys552 [O] |
| Met572 [N] | 2.9 | Glu575 [OE1] |
| Asp573 [N] | 3.7 | Glu575 [OE2] |
| Asn584 [ND2] | 3.1 | Lys467 [O] |
| **Chain A** | **Distribution [Å]** | **Chain B** |
| Glu114 [OE1] | 3.4 | Arg494 [NH1] |
| Glu114 [OE2] | 3.6 | Arg494 [NH1] |
| Asp145 [OD1] | 3.8 | Arg373 [NE] |
| Asp145 [OD1] | 2.6 | Arg373 [NH2] |
| Asp145 [OD1] | 3.7 | Arg494 [NH2] |
| Asp145 [OD2] | 3.6 | Arg373 [NH2] |
| Asp145 [OD2] | 2.7 | Arg494 [NH2] |
