# Supplementary material for: Enzymatic synthesis of l-fucose from l-fuculose using a fucose isomerase from Raoultella sp. and the biochemical and structural analyses of the enzyme
Source: Biotechnol Biofuels. 2019 Dec 5;12:282. doi: 10.1186/s13068-019-1619-0 (PMC6894278; doi:10.1186/s13068-019-1619-0)
Supplement: Supplementary file 9 — Additional file 9: Table S4. Hydrogen bonds and salt bridges on the A–C interface of RdFucI. [file 13068_2019_1619_MOESM9_ESM.docx]

**Additional file 9**

**Table S4** Hydrogen bonds and salt bridges on the A-C interface of *Rd*FucI

| **Chain A**  **(Residue [atom])** | **Distribution [Å]** | **Chain C**  **(Residue [atom])** |
| --- | --- | --- |
| Gly186 [O] | 2.7 | Ser94 [N] |
| Gly186 [N] | 3.8 | Glu95 [OE2] |
| Met185 [O] | 3.3 | Gly93 [N] |
| Ala188 [O] | 3.2 | Gln130 [NE2] |
| Arg303 [NH1] | 2.6 | Tyr92 [OH] |
| Gln302 [OE1] | 2.7 | Arg18 [NH1] |
| Arg363 [NH2] | 2.9 | Thr113 [O] |
| Tyr365 [N] | 3.7 | Tyr139 [OH] |
| Ser367 [OG] | 2.6 | Asp142 [OD1] |
| Arg373 [NH2] | 3.3 | Val143 [O] |
| Tyr440 [O] | 3.4 | Arg18 [NH1] |
| Arg442 [NE] | 3.6 | Met20 [SD] |
| Lys467 [O] | 3.1 | Asn584 [ND2] |
| Lys467 [N] | 3.0 | Ser129 [O] |
| Arg494 [NE] | 2.8 | Thr113 [OG1] |
| Arg494 [O] | 3.5 | Glu114 [N] |
| Tyr518 [OH] | 2.2 | Tyr139 [O] |
| Tyr518 [OH] | 3.4 | Lys158 [NZ] |
| Cys552 [O] | 3.5 | Met572 [N] |
| Tyr579 [OH] | 3.0 | Gly571 [N] |
| Glu575 [OE1] | 2.5 | Met572 [N] |
| Glu575 [OE2] | 2.8 | Asp573 [N] |
| Tyr579 [OH] | 2.6 | Ala569 [O] |
| **Chain A** | **Distribution [Å]** | **Chain B** |
| Arg373 [NH1] | 3.3 | Asp145 [OD2] |
| Arg373 [NH2] | 4.0 | Asp145 [OD2] |
| Arg373 [NH1] | 3.2 | Asp145 [OD1] |
| Arg373 [NH2] | 2.4 | Asp145 [OD1] |
| Arg494 [NH1] | 3.9 | Asp145 [OD1] |
| Arg494 [NH2] | 3.6 | Asp145 [OD1] |
| Arg494 [NH2] | 3.6 | Glu114 [OE1] |
