# Supplementary material for: Enzymatic synthesis of l-fucose from l-fuculose using a fucose isomerase from Raoultella sp. and the biochemical and structural analyses of the enzyme
Source: Biotechnol Biofuels. 2019 Dec 5;12:282. doi: 10.1186/s13068-019-1619-0 (PMC6894278; doi:10.1186/s13068-019-1619-0)
Supplement: Supplementary file 10 — Additional file 10: Table S5. Hydrogen bonds and salt bridges on the A–D interface of RdFucI. [file 13068_2019_1619_MOESM10_ESM.docx]

**Additional file 10**

**Table S5** Hydrogen bonds and salt bridges on the A–D interface of *Rd*FucI

| **Chain A**  **(residue [atom])** | **Distribution [Å]** | **Chain C**  **(residue [atom])** |
| --- | --- | --- |
| Met65 [SD] | 3.4 | Ser176 [OG] |
| Glu95 [OE2] | 3.3 | Gln206 [NE2] |
| Arg77 [NE] | 3.3 | Arg77 [O] |
| Arg77 [NH1] | 3.4 | Gln78 [OE1] |
| Arg77 [NH2] | 3.5 | Ser5 [OG] |
| Lys131 [O] | 2.8 | Lys590 [NZ] |
| Ser176 [OG] | 3.3 | Met65 [SD] |
| Lys204 [NZ] | 3.5 | Ser68 [OG] |
| Gln206 [NE2] | 3.4 | Glu95 [OE2] |
| **Chain A** | **Distribution [Å]** | **Chain C** |
| Lys131 [NZ] | 2.6 | Glu198 [OE2] |
| Glu198 [OE2] | 2.9 | Lys131 [NZ] |
