# Supplementary material for: Enzymatic synthesis of l-fucose from l-fuculose using a fucose isomerase from Raoultella sp. and the biochemical and structural analyses of the enzyme
Source: Biotechnol Biofuels. 2019 Dec 5;12:282. doi: 10.1186/s13068-019-1619-0 (PMC6894278; doi:10.1186/s13068-019-1619-0)
Supplement: Supplementary file 11 — Additional file 11: Table S6. Hydrogen bonds and salt bridges on the A–E interface of RdFucI. [file 13068_2019_1619_MOESM11_ESM.docx]

**Additional file 11**

**Table S6** Hydrogen bonds and salt bridges on the A-E interface of *Rd*FucI

| **Chain A**  **(residue [atom])** | **Distribution [Å]** | **Chain E**  **(residue [atom])** |
| --- | --- | --- |
| Asn195 [OD1] | 2.3 | Lys467 [NZ] |
| Asp209 [OD1] | 3.1 | Met210 [N] |
| Met210 [N] | 3.1 | Asp209 [OD2] |
| Thr211 [O] | 2.9 | Gln308 [NE2] |
| Asp307 [O] | 3.8 | Gln219 [NE2] |
| **Chain A** | **Distribution [Å]** | **Chain E** |
| Glu287 [OE1] | 3.1 | Arg303 [NH2] |
| Glu287 [OE1] | 3.9 | Arg303 [NH1] |
| Glu287 [OE2] | 3.4 | Arg303 [NH2] |
| Glu287 [OE2] | 2.8 | Arg303 [NH1] |
| Arg303 [NH1] | 3.9 | Glu287 [OE1] |
| Arg303 [NH2] | 2.7 | Glu287 [OE1] |
| Arg303 [NH1] | 3.3 | Glu287 [OE2] |
| Arg303 [NH2] | 3.5 | Glu287 [OE2] |
| Lys467 [NZ] | 3.8 | Arg591 [O] |
| Arg591 [O] | 3.9 | Lys467 [NZ] |
