# Supplementary material for: Enzymatic synthesis of l-fucose from l-fuculose using a fucose isomerase from Raoultella sp. and the biochemical and structural analyses of the enzyme
Source: Biotechnol Biofuels. 2019 Dec 5;12:282. doi: 10.1186/s13068-019-1619-0 (PMC6894278; doi:10.1186/s13068-019-1619-0)
Supplement: Supplementary file 12 — Additional file 12: Fig. S6. Structure-based sequence alignment of RdFucI, EcFucI, ApFucI, and SpFucI. [file 13068_2019_1619_MOESM12_ESM.docx]

**Additional file 12**


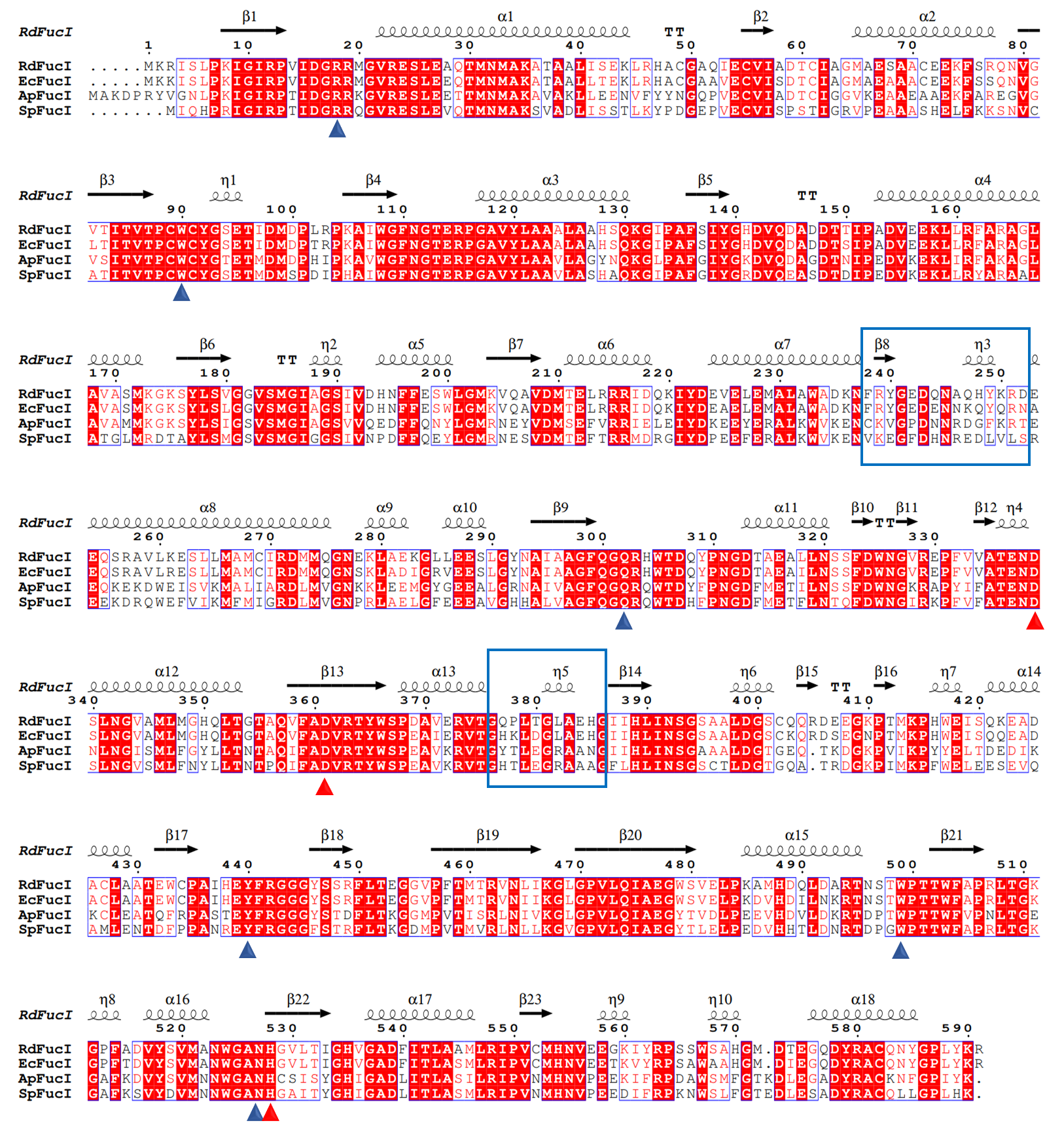


**Fig. S6** Structure-based sequence alignment of *Rd*FucI, *Ec*FucI (UniProt: P69922), *Ap*FucI (C0SSE7), and *Sp*FucI (Q97N97). The metal binding residues are indicated with red triangles. The residues involved in the substrate binding pocket are marked with blue triangles. The unique loop region is indicated by blue boxes.
