# Supplementary material for: Enzymatic synthesis of l-fucose from l-fuculose using a fucose isomerase from Raoultella sp. and the biochemical and structural analyses of the enzyme
Source: Biotechnol Biofuels. 2019 Dec 5;12:282. doi: 10.1186/s13068-019-1619-0 (PMC6894278; doi:10.1186/s13068-019-1619-0)
Supplement: Supplementary file 13 — Additional file 13: Fig. S7. 2Fo-Fc and Fo-Fc electron density maps of metal binding site for (a) RdFucI and (b) RdFucI-Mn2+. [file 13068_2019_1619_MOESM13_ESM.docx]

**Additional file 13**


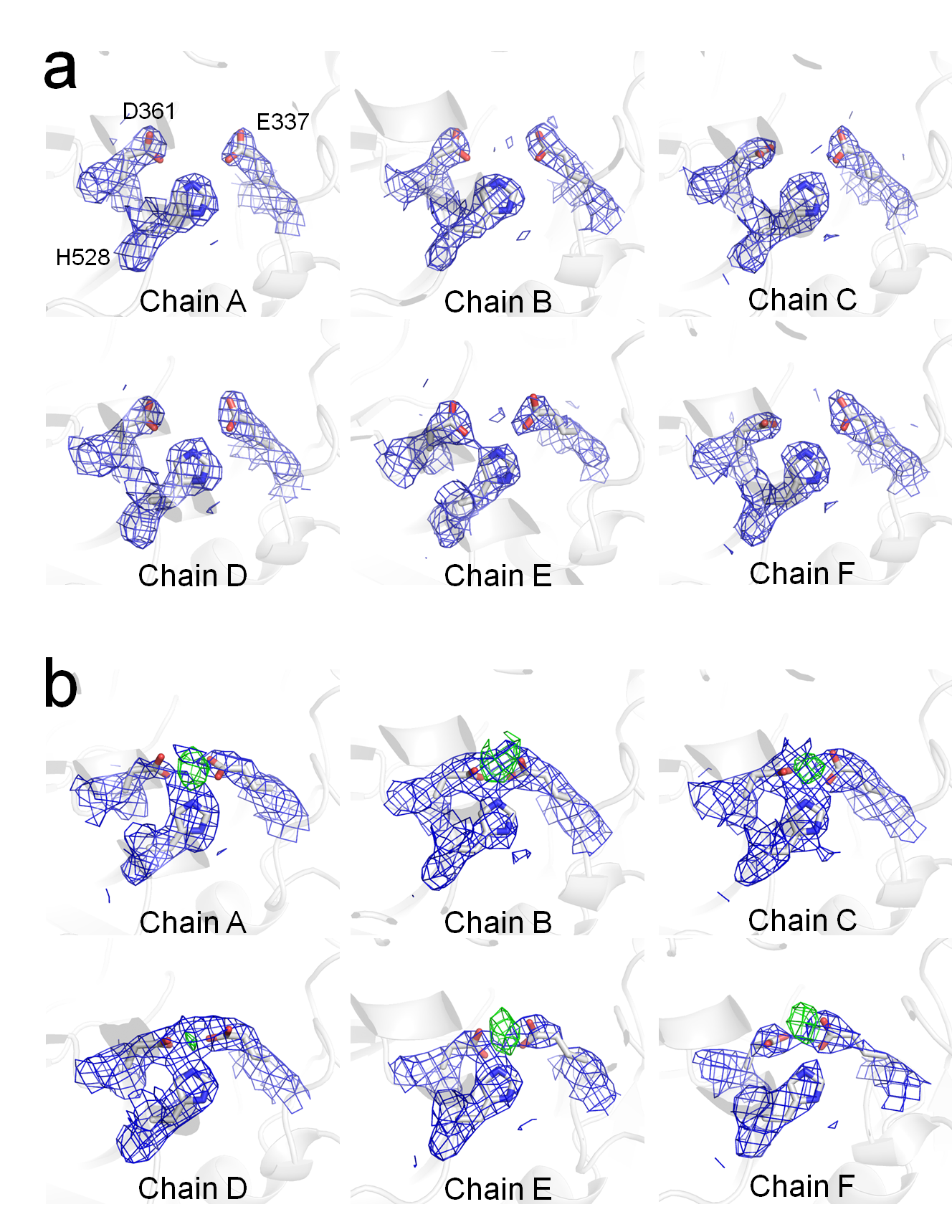


**Fig. S7** 2Fo-Fc (blue mesh, 1.2 σ) and Fo-Fc (green mesh, 5.0 σ) electron density maps of metal binding site for (a) *Rd*FucI and (b) *Rd*FucI-Mn^2+^
